# Design, Synthesis, Molecular Docking, and Preclinical Evaluation of a New Radiolabeled PEG3-Linked FAPI Derivative for Fibroblast Activation Protein Targeting

**DOI:** 10.5812/ijpr-170734

**Published:** 2026-05-23

**Authors:** Mahshid Kiani, Mehdi Akhlaghi, Safura Jokar, Omid Bavi, Hooman Hafezi, Khosrou Abdi, Omid Sabzevari, Saeed Balalaie, Farhad Golmohammadi, Zahra Ghiamaty, Sara Roustaei, Alireza Foroumadi, Davood Beiki

**Affiliations:** 1 Department of Nuclear Pharmacy, Faculty of Pharmacy, Tehran University of Medical Sciences, Tehran, Iran; 2 Research Center for Nuclear Medicine, Tehran University of Medical Sciences, Tehran, Iran; 3 Department of Mechanical Engineering, Shiraz University of Technology, Shiraz, Iran; 4 Department of Toxicology and Pharmacology, Faculty of Pharmacy, Tehran University of Medical Sciences, Tehran, Iran; 5 Toxicology and Poisoning Research Center, Tehran University of Medical Sciences, Tehran, Iran; 6 Peptide Chemistry Research Institute, K. N. Toosi University of Technology, Tehran, Iran; 7 Department of Medicinal Chemistry, Faculty of Pharmacy, Tehran University of Medical Sciences, Tehran, Iran

**Keywords:** Cancer-associated Fibroblasts, Gallium-68, Molecular Imaging, Molecular Dynamics, PET Imaging, Radiolabeling

## Abstract

**Background:**

Fibroblast activation protein (FAP) is a promising molecular target for cancer theranostic applications. However, current fibroblast activation protein inhibitors (FAPIs) have limitations, including rapid clearance and limited tumor retention.

**Objectives:**

This study aimed to develop a novel PEG_3_-linked FAPI derivative, [^68^Ga]Ga-FAPI-MKG, with enhanced tumor accumulation and retention. FAPI-MKG was prepared by incorporating a PEG_3_ linker into the FAPI-04 structure to optimize its pharmacokinetic profile.

**Methods:**

The compound was synthesized via an 11-step route starting from quinine sulfate and radiolabeled with gallium-68. In vitro studies included determination of lipophilicity (Log P) and stability assays in saline and human serum albumin. Preclinical evaluation in BALB/c mice bearing CT-26 tumors included biodistribution, blocking studies, and PET/CT imaging. Molecular docking and molecular dynamics simulations were performed to provide mechanistic insights into binding interactions.

**Results:**

[^68^Ga]Ga-FAPI-MKG was successfully synthesized with high radiochemical purity (> 98%) and a molar activity of 414.79 mCi/μmol. It demonstrated moderate hydrophilicity (Log P = -3.26 ± 0.18) compared with the reference radiotracer, [^68^Ga]Ga-FAPI-46 (-3.58 ± 0.29), and high stability (RCP > 90% after 120 minutes). In vivo studies showed significantly higher tumor uptake (7.18 ± 0.56% ID/g at 60 minutes; 3.20 ± 0.11% ID/g at 120 minutes) and prolonged retention compared with the reference radiotracer, along with dual hepatobiliary and renal excretion pathways. Blocking studies confirmed FAP-specific uptake. Computational analyses indicated strong binding energy (-9.8 kcal/mol) and optimized electrostatic interactions with FAP. The strategic incorporation of a PEG_3_ linker into [^68^Ga]Ga-FAPI-MKG significantly improved tumor accumulation, extended tumor retention, and increased the tumor-to-background ratio.

**Conclusions:**

These findings suggest that [^68^Ga]Ga-FAPI-MKG may be a promising candidate for clinical translation for imaging and theranostics of FAP-expressing cancers.

## 1. Background

Cancer-associated fibroblasts (CAFs) constitute a prominent stromal population within the tumor microenvironment (TME). Upon activation by tumor-associated signals, CAFs promote tumor progression, including growth, angiogenesis, invasion, and metastasis, through the secretion of extracellular matrix components, cytokines, and growth factors. Therefore, understanding CAFs and other components of the TME is essential for developing effective diagnostic and therapeutic agents, including radiopharmaceuticals (1-4).

Fibroblast activation protein (FAP) is a 170-kDa transmembrane serine protease and a key hallmark and functional driver of CAFs. Since its first identification in 1986, FAP has been shown to be selectively overexpressed under pathological conditions and to contribute critically to tumor progression by facilitating extracellular matrix remodeling, tumor invasion, metastasis, and immunosuppressive activity. Its highly specific overexpression on CAFs, in contrast to its minimal expression in healthy tissues, establishes FAP as a key molecular target for precise radioligand-based theranostic strategies (4-6). Recently, considerable advances have been achieved in the development of quinoline-based FAPIs, including FAPI-02, FAPI-04, and FAPI-46 (5, 7, 8). These small molecules, often incorporating the UAMC1110 pharmacophore, demonstrate high selectivity for FAP and represent common structural motifs for new targeted radiotracers. The clinical translation of these compounds has been promising. FAPI-based PET/CT imaging has shown clear advantages over the traditional standard, [^18^F]FDG PET/CT, in identifying primary tumors and metastases across different malignancies. These benefits include higher tumor uptake, increased sensitivity, improved tumor-to-background imaging contrast, and a higher overall tumor detection rate (8, 9). Accordingly, these quinoline-based inhibitors have been successfully radiolabeled with both diagnostic (eg, ^68^Ga and ^18^F) and therapeutic (eg, ^177^Lu and ^90^Y) radionuclides, enabling their application in cancer theranostics and radioligand therapy (5, 10). Although current FAPI radiotracers demonstrate favorable imaging characteristics for diagnosis, their rapid blood clearance, suboptimal tumor targeting, and short tumor retention time significantly limit their therapeutic efficacy in targeted radioligand therapy. To date, numerous modified FAPI derivatives have been introduced to overcome the pharmacokinetic limitations of earlier generations (11-13).

## 2. Objectives

In this study, we developed a novel radiotracer, [^68^Ga]Ga-FAPI-MKG, by incorporating a PEG_3_ linker into the FAPI-04 structure to enhance tumor retention and targeting. PEGylated compounds exhibit favorable pharmacokinetic properties, including modulated hydrophobicity, a prolonged plasma half-life, reduced immunogenicity, and improved tumor penetration (14, 15). FAPI-MKG was synthesized and radiolabeled with [^68^Ga]GaCl_3_ to produce [^68^Ga]Ga-FAPI-MKG, and was evaluated using a comprehensive preclinical strategy. This integrated approach included in vitro stability studies, biodistribution studies, blocking experiments, and PET/CT imaging in BALB/c CT-26 tumor-bearing mice. Molecular docking studies and molecular dynamics simulations were also performed to assess the binding modes and interaction energies with the FAP receptor, providing a mechanistic rationale for the observed targeting efficacy.

## 3. Methods

### 3.1. Material and Methods

All reagents and solvents were obtained from Sigma-Aldrich (Munich, Germany) and Merck (Darmstadt, Germany) and used as received. Synthesized compounds were characterized by ^1^H NMR spectroscopy using a JNM-ECS spectrophotometer (JEOL, Tokyo, Japan) and by mass spectrometry using a Synapt G1 HDMS system (Q-TOF analyzer, ESI mode). Chemical purity was determined by high-performance liquid chromatography (HPLC) using a Shimadzu CL-20AVPV system equipped with an SPD-20A UV detector (λ = 54 nm) and a Kromasil 100 - 5 column (250 × 4.6 mm). [^68^Ga]GaCl_3_ was prepared by eluting a ^68^Ge/^68^Ga generator (Pars Isotope Co., Karaj, Iran; 110 MBq/day). Radiochemical purity was assessed by ITLC-SG (Merck) using a radio-TLC scanner (RAYTEST, Straubenhardt, Germany) and confirmed by radio-reverse-phase HPLC (RP-HPLC) using an Agilent 1200 system with an in-line gamma detector (RAYTEST). The CT-26 murine colon carcinoma and NIH-3T3 murine embryonic fibroblast cell lines were obtained from the Pasteur Institute of Iran (Tehran, Iran). PET/CT imaging was performed on a Biograph 6 system (Siemens Medical Solutions, Erlangen, Germany), and images were reconstructed using Syngo software (Siemens Healthineers). Molecular modeling, including docking and dynamics simulations, was performed using AutoDock Vina, NAMD 3.0, and VMD 1.9.4, supported by additional computational tools (16, 17). All compounds exhibited > 95% purity, as determined by HPLC analysis.

Detailed synthetic procedures and characterization of the intermediate and final products, including their analytical spectra, are provided in the Supplementary File.

### 3.2. FAPI-MKG Synthesis and Characterization

FAPI-MKG was synthesized using an 11-step procedure. The synthetic route involved the stepwise assembly of the molecular scaffold, incorporating a quinine-based core, a PEG_3_ linker, and a 1,4,7,10-tetraazacyclododecane-1,4,7,10-tetraacetic acid (DOTA) moiety. Each intermediate and the final compound were purified and characterized using standard analytical techniques, including ^1^H NMR spectroscopy and mass spectrometry. Chemical purity was measured by HPLC (see Supplementary File).

### 3.3. Radiolabeling of FAPI-MKG With Gallium-68

For the preparation of [^68^Ga]Ga-FAPI-MKG, a fresh solution of [^68^Ga]GaCl_3_ was obtained by eluting a sterile ^68^Ge/^68^Ga generator with 0.1 M HCl (3 mL). The eluate (9.25 mCi per 3 mL) was added to a reaction vial containing 25 μg of the FAPI-MKG precursor. The pH of the reaction mixture was adjusted to approximately 4.0 with 320 μL of sodium acetate buffer (1 M, pH = 7.0), and the mixture was then incubated at 95 - 100°C for 12 minutes. After incubation, the reaction mixture was cooled to room temperature and loaded onto a preconditioned Sep-Pak C18 cartridge. Unbound radioactivity and impurities were removed by washing with normal saline, and the purified product was eluted with a 1:1 (v/v) ethanol:water solution. The final solution was passed through a 0.22-μm sterile filter (Merck Millipore, Darmstadt, Germany) into a sterile vial. For animal studies, the product was diluted with NaCl, and the ethanol content was reduced to below 10% (6, 18, 19). Radiochemical purity (RCP) was determined using two complementary methods: RP-HPLC and ITLC. RP-HPLC analysis was performed on an Agilent 1200 system with a gamma detector fitted with a Thermo Fisher C18 column (150 × 3 mm, 3 μm). Separation was achieved at a flow rate of 1.0 mL/min using a 40-minute gradient of water and acetonitrile, each containing 0.1% trifluoroacetic acid. ITLC analysis was conducted on silica gel-impregnated glass fiber (ITLC-SG) strips, using a mixture of 1 M ammonium acetate and methanol (1:1, v/v) as the mobile phase. Radioactivity distribution for both methods was quantified using a radio-TLC scanner (18).

### 3.4. in vitro Stability Studies

The in vitro stability of [^68^Ga]Ga-FAPI-MKG was evaluated by incubating aliquots of the radiotracer (100 μL, 3.7 MBq) in 1.0 mL of two different media: NaCl (pH = 5.5) and a human serum albumin (HSA) solution. The radiochemical purity of the samples incubated at 37°C was assessed at 30, 60, and 120 minutes after incubation using ITLC, according to the method described above (13, 20).

### 3.5. Partition Coefficient (Log P) Measurement

The lipophilicity of [^68^Ga]Ga-FAPI-MKG was assessed by determining its partition coefficient (Log P) in an n-octanol/PBS buffer system. An aliquot of the radiotracer (100 μL) was added to a vial containing presaturated n-octanol (1.0 mL) and normal saline (1.0 mL), vortexed for 5 minutes to reach equilibrium, and then centrifuged at 5000 rpm for 10 minutes at room temperature to separate the phases. Aliquots (500 μL) were collected from each phase, and the radioactivity was quantified using a calibrated dose calibrator (Capintec CRC-25, Florham Park, NJ, USA). The partition coefficient (Log P) was calculated from the ratio of radioactivity between the n-octanol and aqueous phases. All experiments were performed in triplicate, and the results are reported as mean ± standard deviation (18, 20).

### 3.6. Biodistribution Studies and PET/CT Imaging

Male BALB/c mice (6 - 8 weeks old, 20 - 25 g) were obtained from the Institute of Biochemistry and Biophysics (Tehran, Iran) and housed under a 12-hour light/dark cycle at 20 - 22°C. For tumor xenograft establishment, CT-26 colon carcinoma cells (1 × 10^6^ cells) and NIH-3T3 cells (2 × 10^6^ cells), each in 150 μL PBS, were subcutaneously injected into the animals' right shoulder. Biodistribution studies and PET/CT scintigraphy were conducted 10 - 14 days after tumor inoculation (6, 18). Tumor-bearing mice were randomly allocated into 3 groups: 1) [^68^Ga]Ga-FAPI-MKG (100 μL, 3.7 MBq), 2) [^68^Ga]Ga-FAPI-46 (100 μL, 3.7 MBq; reference group), and 3) a blocking group (pretreated with cold FAPI-46). Mice were intravenously injected via the tail vein and euthanized at different time points (30, 60, 90, and 120 minutes). The selected organs were removed, washed with cold saline, weighed, and radioactivity was measured using a gamma counter. Biodistribution results were expressed as the percentage injected dose per gram of tissue (%ID/g, mean ± SD). For blocking studies, a 300-fold molar excess of cold FAPI-46 was administered intravenously 30 minutes before [^68^Ga]Ga-FAPI-MKG injection to evaluate receptor-specific binding (6, 18, 21). Whole-body PET/CT imaging was performed in all 3 experimental groups using a PET/CT scanner (Biograph 6, Siemens Medical Solutions, Erlangen, Germany). Mice were intravenously injected with the radiotracers and anesthetized with a ketamine/xylazine mixture. Animals were positioned prone and scanned at 30, 60, 90, and 120 minutes after injection. CT scans were acquired for anatomical localization and attenuation correction (acquisition time: 20 seconds; 80 kV; 150 mAs; 1.25-mm spatial resolution). PET/CT images were reconstructed using a filtered back-projection algorithm and subsequently coregistered with CT images for anatomical localization and quantitative analysis (18, 19, 21). Statistical analyses were performed using 1-way ANOVA and Student t test in GraphPad Prism software.

## 4. Results

### 4.1. Synthesis and Radiolabeling of FAPI-MKG With ^68^Ga

**Figure 1.**
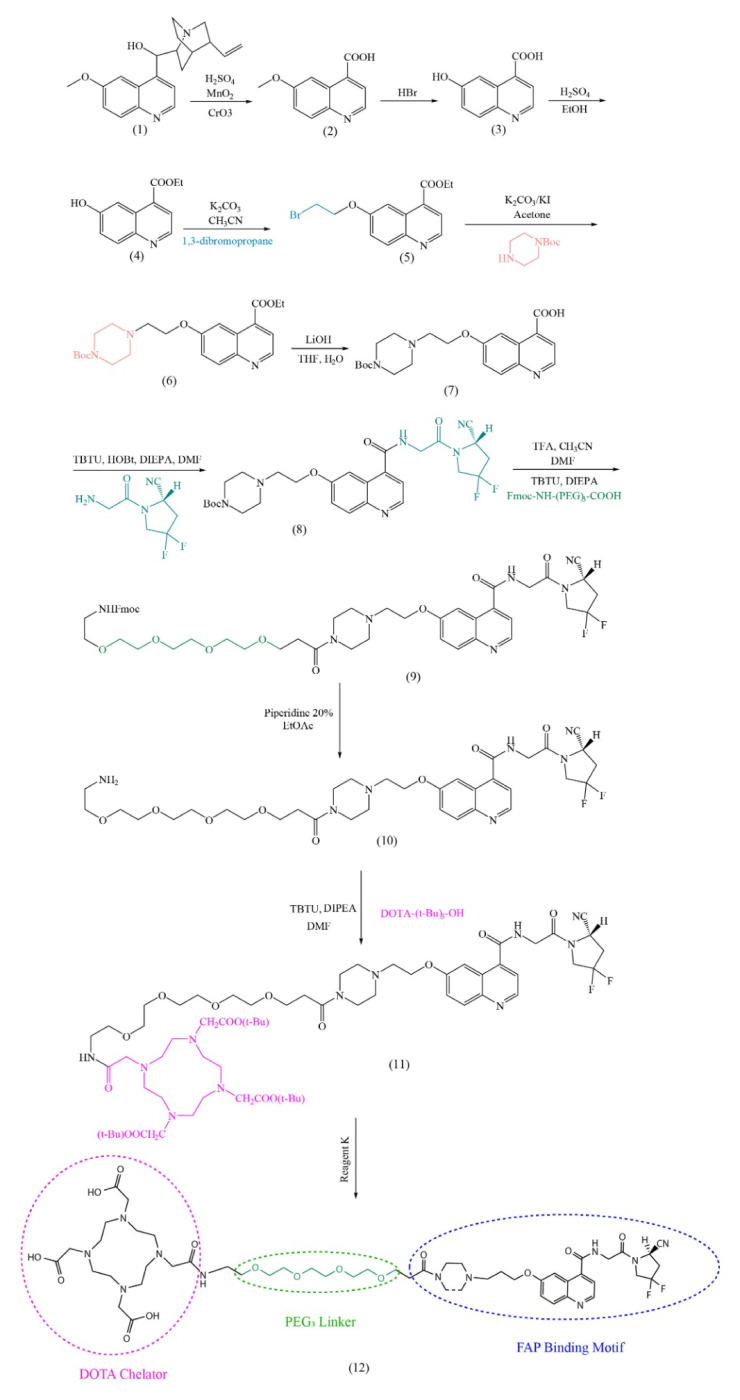
Synthesis pathway for the preparation of FAPI-MKG. The scheme illustrates the stepwise construction of the quinine-based small-molecule scaffold, incorporation of the PEG3 linker, and final conjugation with the 1,4,7,10-tetraazacyclododecane-1,4,7,10-tetraacetic acid (DOTA) chelator.

FAPI-MKG was successfully synthesized via an 11-step procedure (Figure 1). The synthetic route involved the stepwise assembly of the molecular scaffold, incorporating a quinine-based core, a PEG_3_ linker, and a DOTA moiety. Each intermediate and the final compound were purified and characterized using standard analytical techniques, including ^1^H NMR spectroscopy and mass spectrometry. Chemical purity was assessed by HPLC.

HPLC purification of the final product yielded a white powder with a purity of greater than 98%. As presented in the Supplementary File (Figure S21B in the Supplementary File), the HPLC peak at 21.08 minutes corresponded to FAPI-MKG. HR-MS analysis (TOF-MS-ES^+^) confirmed the expected molecular structure of FAPI-MKG, with a calculated molecular weight of 1120.2166 g/mol and peaks of [M+H]^+^ and [M+2H]^+^/2 as the main dissociation products at m/z = 560.6307 and m/z = 1119.5412, respectively (Supplementary File, Figure S21A in the Supplementary File).

[^68^Ga]Ga-FAPI-MKG was successfully prepared with an RCP of approximately 99%. As shown in the HPLC chromatogram (Figure S22A in the Supplementary File), the retention times for free ^68^Ga and [^68^Ga]Ga-FAPI-MKG were 1.45 and 22.98 minutes, respectively. ITLC analysis further confirmed the radiochemical identity, with retention factors of 0.1 for colloidal forms of ^68^Ga and 0.5 for [^68^Ga]Ga-FAPI-MKG (Figure S22B in the Supplementary File).

### 4.2. in vitro Stability Studies

**Figure 2.**
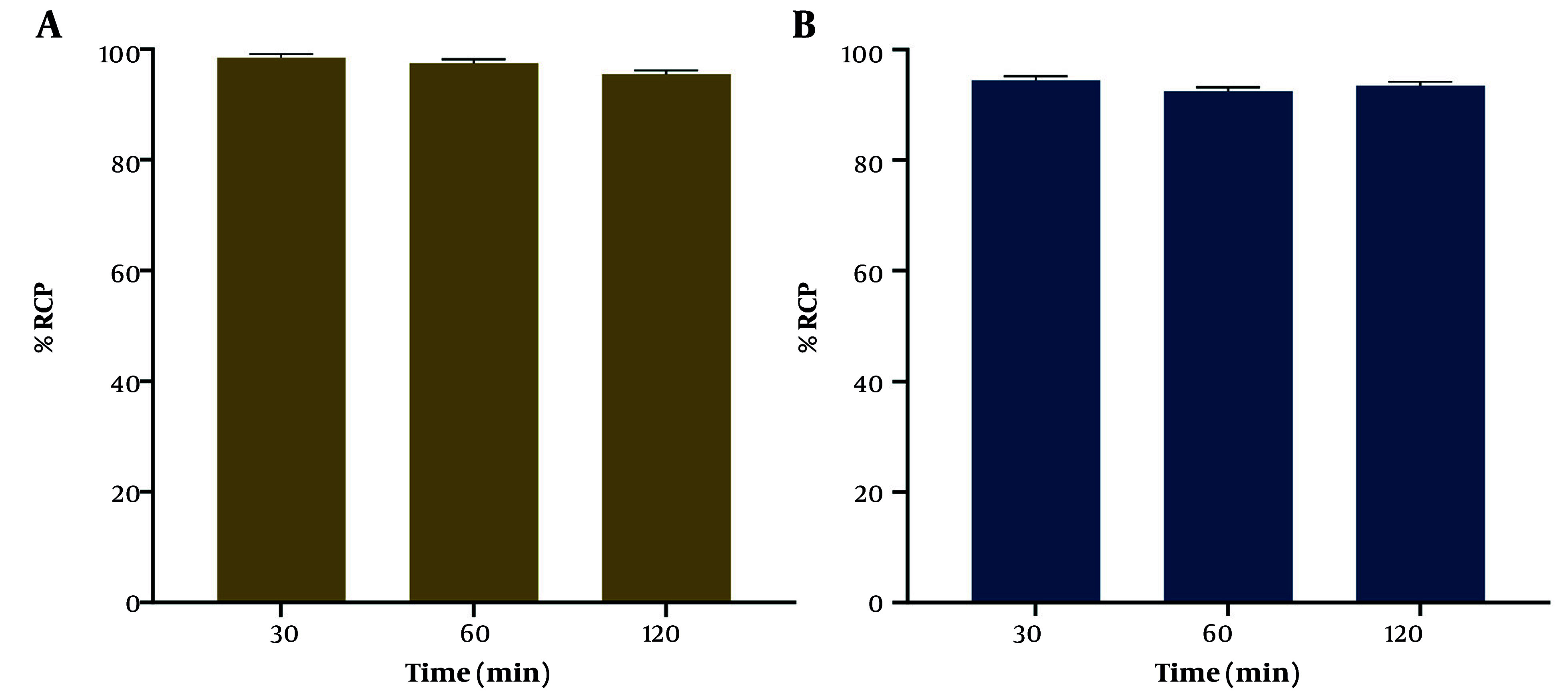
Analysis of the in vitro stability studies of [^68^Ga]Ga-FAPI-MKG in (A) normal saline and (B) HSA at 37°C up to 120 minutes (mean ± SD, n = 3).

The in vitro stability of [^68^Ga]Ga-FAPI-MKG was evaluated under physiological conditions by incubating the radiotracer in normal saline and HSA at 37°C. Radiochemical purity was assessed at 30, 60, and 120 minutes after incubation using ITLC-SG with a methanol/ammonium acetate 1:1 (v/v) mobile phase. The radiotracer showed high stability, retaining over 95% radiochemical purity in both normal saline and HSA at 120 minutes (Figure 2).

### 4.3. Partition Coefficient Studies (Log P)

The measured Log P values were -3.26 ± 0.18 for [^68^Ga]Ga-FAPI-MKG and -3.58 ± 0.29 for [^68^Ga]Ga-FAPI-46 (mean ± SD, n = 3). These results indicate that both radiotracers are highly hydrophilic. However, [^68^Ga]Ga-FAPI-MKG exhibited slightly higher lipophilicity than [^68^Ga]Ga-FAPI-46.

### 4.4. Biodistribution Studies

**Figure 3.**
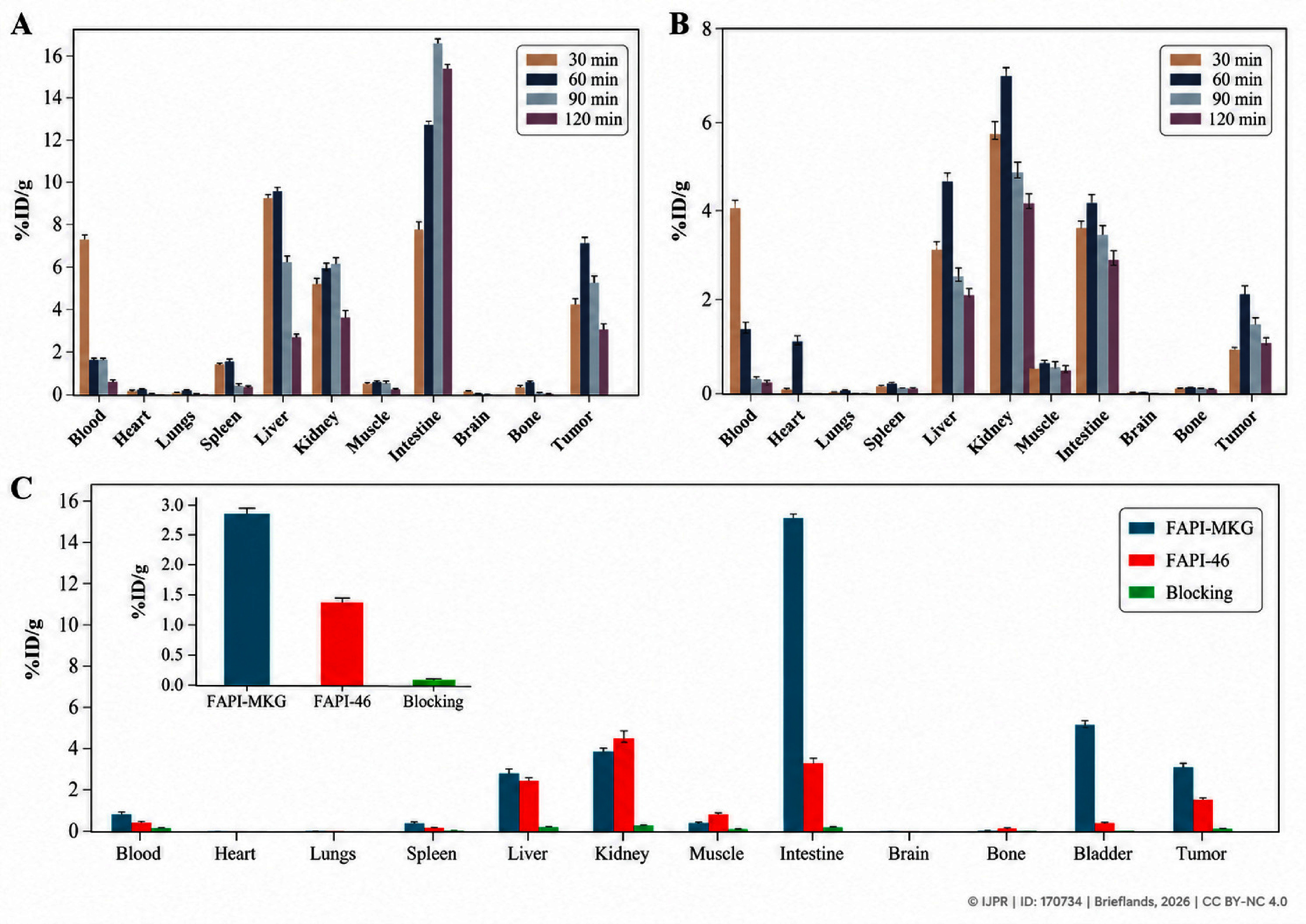
Results of the biodistribution studies of (A) [68Ga]Ga-FAPI-MKG and (B) [68Ga]Ga-FAPI-46 (reference group) in tumor-bearing mice at 30, 60, 90, and 120 minutes after injection. (C) Blocking group (pretreated with cold FAPI-46) at 120 minutes after injection (as %ID/g, mean ± SD, n = 3).

Biodistribution studies of [^68^Ga]Ga-FAPI-MKG and the reference radiotracer [^68^Ga]Ga-FAPI-46 were performed in tumor-implanted mice at 30, 60, 90, and 120 minutes after injection. As shown in Figures 3A and B, [^68^Ga]Ga-FAPI-MKG demonstrated higher tumor accumulation (7.18 ± 0.056% ID/g at 60 minutes) and prolonged lesion retention than [^68^Ga]Ga-FAPI-46 (2.175 ± 0.078% ID/g at 60 minutes). Both radiotracers initially showed high uptake in nontarget tissues, including the liver, kidneys, bladder, intestine, and spleen, followed by a gradual decrease over time. Blood uptake of [^68^Ga]Ga-FAPI-MKG decreased rapidly from 30 minutes (7.40 ± 0.05% ID/g) to 120 minutes (0.50 ± 0.07% ID/g), whereas [^68^Ga]Ga-FAPI-46 exhibited slightly slower clearance (4.07 ± 0.07% ID/g at 30 minutes and 0.21 ± 0.02% ID/g at 120 minutes).

**Figure 4.**
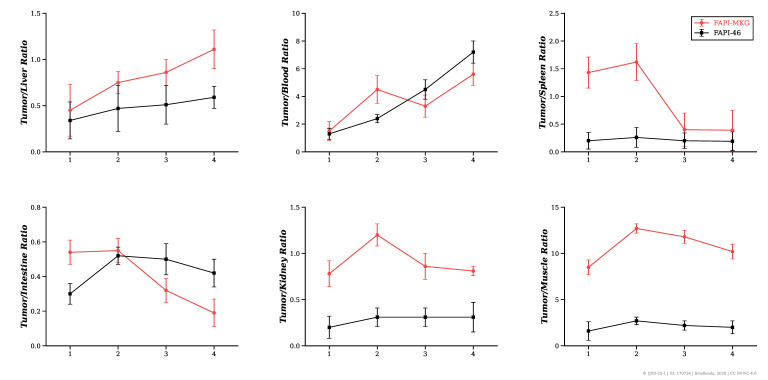
The calculated tumor-to-organ uptake ratios of [^68^Ga]Ga-FAPI-MKG and [68Ga]Ga-FAPI-46 at 30, 60, 90, and 120 minutes after injection (mean ± SD, n = 3).

To evaluate the FAP-specific binding of [^68^Ga]Ga-FAPI-MKG, blocking was performed using unlabeled FAPI-46. Figure 3C shows that tumor accumulation of [^68^Ga]Ga-FAPI-MKG in the blocked group (0.07 ± 0.1% ID/g) was significantly lower than that in the unblocked group (3.20 ± 0.11% ID/g) at 120 minutes. Tumor-to-organ uptake ratios for [^68^Ga]Ga-FAPI-MKG and [^68^Ga]Ga-FAPI-46, calculated for the kidneys, muscle, intestine, liver, and blood at all time points, are shown in Figure 4. [^68^Ga]Ga-FAPI-MKG demonstrated higher tumor-to-kidney, tumor-to-muscle, and tumor-to-liver ratios than [^68^Ga]Ga-FAPI-46 across all time points. However, the tumor-to-blood and tumor-to-intestine ratios for [^68^Ga]Ga-FAPI-MKG decreased after 60 minutes compared with those for [^68^Ga]Ga-FAPI-46.

### 4.5. Imaging Studies

**Figure 5.**
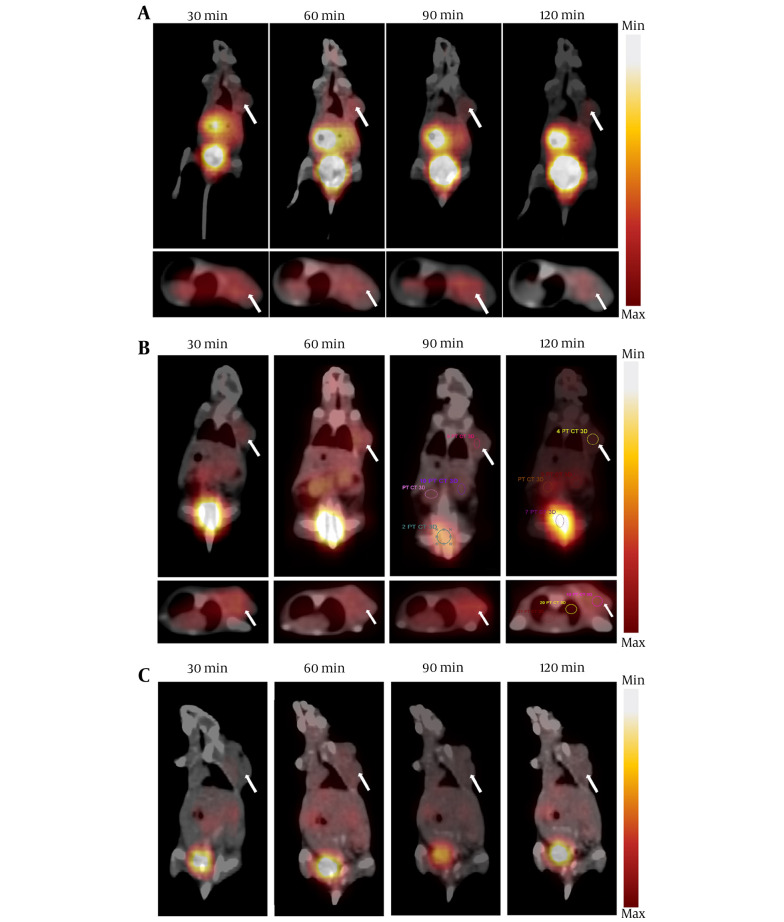
The fused PET/CT images, in coronal and axial views, taken at 30, 60, 90, and 120 minutes after injection of (A) [^68^Ga]Ga-FAPI-MKG, (B) [^68^Ga ]Ga-FAPI-46, and (C) the blocking group. The arrows indicate the tumor site.

In vivo PET/CT scintigraphy was performed in tumor-bearing BALB/c mice in the different groups after intravenous injection of 3.7 MBq of the radiotracers. Scans were acquired at 30, 60, 90, and 120 minutes after injection in both coronal and axial views. Figure 5 shows the fused PET/CT images for [^68^Ga]Ga-FAPI-MKG, [^68^Ga]Ga-FAPI-46 (reference group), and the blocking group at each time point. As shown in Figures 5A and B, both radiotracers, [^68^Ga]Ga-FAPI-MKG and [^68^Ga]Ga-FAPI-46, exhibited significant uptake in the inoculated tumor in the right shoulder of the mice. [^68^Ga]Ga-FAPI-46 demonstrated predominant renal excretion, whereas [^68^Ga]Ga-FAPI-MKG showed altered clearance kinetics. In the blocking group (Figure 5C), tumor uptake of [^68^Ga]Ga-FAPI-MKG was significantly reduced at all time points, confirming the specificity of the radiotracer for FAP-positive tumors.

## 5. Discussion

The FAP is selectively overexpressed on CAFs within the TME of various cancers (22). FAP has emerged as a highly promising theranostic target because of its multiple roles in tumor progression, including extracellular matrix remodeling, tumor growth, invasion, angiogenesis, therapy resistance, and immunosuppression within the TME. PET imaging using FAPIs provides a highly sensitive and specific noninvasive modality that enables accurate diagnosis, staging, treatment monitoring, and therapy evaluation in FAP-expressing tumors (5, 23). First- and second-generation DOTA-conjugated FAPIs, such as FAPI-02, FAPI-04, and FAPI-46, have been introduced for clinical studies. These compounds contain a 6-hydroxy and 6-amino quinoline core (UAMC-1110) in their chemical structures. Despite their promising diagnostic performance, FAPI-based radiotracers face significant pharmacokinetic limitations that restrict their therapeutic application. These include rapid blood clearance, short intratumoral retention times, and moderate tumor uptake, which remain key challenges for the clinical translation of targeted radioligand therapy. Structure-activity relationship studies have systematically investigated modifications of the pharmacophore, linkers, and chelators to optimize the pharmacokinetic profiles of various radiotracers (24). A variety of pharmacokinetic-modifying linkers, such as polyethylene glycol, aminohexanoic acid, amino acids, hydrocarbon chains, peptides, hexaethylene glycol, and para-aminomethylaniline-diglycolic acid, have been incorporated to enhance tumor targeting and clearance profiles. Studies demonstrate that PEGylation strategies, whether through covalent or noncovalent conjugation of PEG to drug structures, including proteins, small molecules, peptides, nucleic acids, and antibodies, improve target-to-background ratios by enhancing complex stability, increasing tumor uptake, reducing nonspecific accumulation, and prolonging circulation time (25, 26).

Based on this rationale, FAPI-MKG was synthesized by incorporating a PEG_3_ linker into the FAPI-04 scaffold to enhance tumor targeting and pharmacokinetic properties. The compound was successfully synthesized through an 11-step synthetic route starting from quinine sulfate as the initial precursor, achieving high chemical purity (> 98%) (Figure S21 in the Supplementary File). Radiolabeling of FAPI-MKG was performed using freshly eluted ^68^GaCl_3_ under optimized conditions (95 - 100°C, 12 minutes, and pH = 4.0), with a molar activity of 414.79 mCi/mmol and radiochemical purity of more than 98% (Figure S22 in the Supplementary File). The partition coefficients (Log P) of [^68^Ga ]Ga-FAPI-MKG and the reference radiotracer [^68^Ga ]Ga-FAPI-46 were determined to be -3.26 ± 0.18 and -3.58 ± 0.29, respectively. These results indicate that both radiotracers are highly hydrophilic, although [^68^Ga ]Ga-FAPI-MKG exhibits slightly lower hydrophilicity than the reference. The in vitro stability of [^68^Ga ]Ga-FAPI-MKG was evaluated in normal saline and HSA at 37°C at various time points using ITLC. [^68^Ga ]Ga-FAPI-MKG demonstrated high stability, retaining radiochemical purity of more than 90% over the incubation period (Figure 2). Biodistribution was assessed in BALB/c tumor-bearing mice across 3 experimental groups: 1) [^68^Ga ]Ga-FAPI-MKG, 2) [^68^Ga ]Ga-FAPI-46 (reference group), and 3) the blocked group (pretreated with unlabeled FAPI-MKG) (Figures 3 and 4). At 30 minutes after injection, [^68^Ga ]Ga-FAPI-MKG exhibited significantly higher blood activity (7.395 ± 0.049% ID/g) than [^68^Ga ]Ga-FAPI-46 (4.07 ± 0.071% ID/g), indicating an approximately 2-fold longer circulation time for [^68^Ga ]Ga-FAPI-MKG. Blood activity decreased to 0.5 ± 0.071% ID/g for [^68^Ga ]Ga-FAPI-MKG and 0.205 ± 0.021% ID/g for the reference radiotracer at 120 minutes after injection, confirming sustained clearance in both groups. A statistically significant difference in tumor accumulation kinetics was observed between the 2 radiotracers (P < 0.001). The highest tumor uptake of [^68^Ga ]Ga-FAPI-MKG (7.18 ± 0.056% ID/g) and [^68^Ga ]Ga-FAPI-46 (2.175 ± 0.078% ID/g) occurred 60 minutes after injection, demonstrating a 1.5-fold higher uptake for [^68^Ga ]Ga-FAPI-MKG than for the reference tracer. By 120 minutes, [^68^Ga ]Ga-FAPI-MKG (3.20 ± 0.113% ID/g) maintained 5.3-fold higher tumor accumulation than [^68^Ga ]Ga-DOTA-FAPI-46 (1.36 ± 0.056% ID/g) (P < 0.0001), confirming improved tumor targeting and prolonged tumor retention attributable to PEG_3_ linker modification. Both radiotracers showed prominent renal uptake due to their hydrophilic nature (Log P: -3.26 vs -3.58). However, [^68^Ga ]Ga-FAPI-MKG, with its relatively lower hydrophilicity, demonstrated dual hepatobiliary and renal excretion, leading to substantial activity accumulation in the liver, intestine, and kidneys (Figures 3A and 5A). This shift in excretion kinetics is likely attributable to structural modifications in FAPI-MKG, specifically the incorporation of the PEG_3_ linker, which modulates hydrophilicity and facilitates hepatobiliary clearance alongside renal excretion, as previously documented for PEGylated radiopharmaceuticals (14, 15). The amphiphilic nature of the PEGylated linker enhances passive diffusion across hepatocyte membranes, enabling biliary elimination while retaining sufficient hydrophilicity for partial renal clearance. This dual excretion pathway could explain the observed hepatic accumulation and prolonged circulation of [^68^Ga ]Ga-FAPI-MKG compared with [^68^Ga ]Ga-FAPI-46. Blocking studies demonstrated that preadministration of nonlabeled FAPI-46 led to a significant reduction (P < 0.0001) in tumor uptake of [^68^Ga ]Ga-FAPI-MKG compared with the nonblocked group (Figures 3C and 5C). This finding confirms that tumor accumulation of [^68^Ga ]Ga-FAPI-MKG is primarily mediated through FAP-specific binding mechanisms, with negligible nonspecific uptake. Tumor-to-blood and tumor-to-muscle ratios are critical parameters for evaluating tumor-to-background contrast and determining the optimal imaging time window. The maximum tumor-to-muscle ratio for [^68^Ga ]Ga-FAPI-MKG was calculated to be 12.82 ± 0.22 at 60 minutes after injection (Figure 4), indicating significantly enhanced tumor uptake and minimal nontarget background activity. This high ratio corresponds to favorable tumor-to-background contrast and identifies 60 minutes after injection as the optimal imaging time point, resulting in superior resolution and image quality in PET/CT scans (Figure 5A). As shown in Figure 5, tumor sites were clearly visualized with both [^68^Ga ]Ga-FAPI-MKG and [^68^Ga ]Ga-FAPI-46, whereas minimal uptake was observed in the blocking group, confirming the FAP specificity of both radiotracers. The PET/CT images visually confirmed the excretory pathways quantified in the biodistribution studies: [^68^Ga ]Ga-FAPI-MKG showed dual hepatobiliary and renal excretion, whereas [^68^Ga ]Ga-FAPI-46 showed predominantly renal excretion. This result demonstrates good agreement between qualitative imaging and quantitative biodistribution data and highlights how structural modifications, including the PEG_3_ linker in FAPI-MKG, alter pharmacokinetic profiles.

The computational results (see Supplementary File) align with the in vivo biodistribution and imaging data, confirming that structural modification with the PEG_3_ linker in FAPI-MKG enhances tumor targeting and pharmacokinetic profiles. Consistency across computational, in vitro, and in vivo results underscores the reliability of the findings and provides a mechanistic basis for the improved performance of FAPI-MKG.

### 5.1. Conclusions

This study successfully designed, synthesized, and evaluated a novel FAP-targeted radiotracer, [^68^Ga ]Ga-FAPI-MKG. The rational incorporation of a PEG_3_ linker into the FAPI scaffold proved to be a critical structural modification, significantly improving the pharmacokinetic profile compared with the reference radiotracer, [^68^Ga ]Ga-FAPI-46. The results demonstrated that [^68^Ga ]Ga-FAPI-MKG exhibits high in vitro stability, enhanced tumor targeting, and prolonged intratumoral retention. Its dual hepatobiliary and renal excretion pathways may contribute to a favorable tumor-to-background ratio, resulting in superior PET/CT image contrast. Specific FAP-mediated uptake was confirmed by blocking studies and supported by molecular docking and dynamics simulations, which revealed more stable complexes with FAP through optimized electrostatic interactions. Collectively, these findings support further clinical translation of [^68^Ga ]Ga-FAPI-MKG as a promising theranostic radiotracer for FAP-expressing tumors.

ijpr-25-1-170734-s001.pdf

## Data Availability

The data presented in this study are uploaded during submission as a supplementary file and are openly available for readers upon request
