# Supplementary material for: Design, Synthesis, Molecular Docking, and Preclinical Evaluation of a New Radiolabeled PEG3-Linked FAPI Derivative for Fibroblast Activation Protein Targeting
Source: Iran J Pharm Res. 2026 May 23;25(1):e170734. doi: 10.5812/ijpr-170734 (PMC13389375; doi:10.5812/ijpr-170734)
Supplement: ijpr-25-1-170734-s001.pdf [file ijpr-25-1-170734-s001.pdf]

Supplementary Material

S1. FAPI-MKG Synthesis and Characterization

Synthesis of 4-carboxylic acid-6-methoxy quinoline (quinine acid) (2). Quinine sulfate (1) (0.9 mmol) was dissolved in 12 mL of 10% H_2SO_4. Manganese dioxide (MnO_2) was added to the solution, and the reaction mixture was connected to a reflux condenser. A solution of chromium trioxide (CrO_3; 14.30 mmol in 3 mL water) was added dropwise under continuous stirring. The reaction was refluxed for 3 hours. Subsequently, 126 mL of water and 28 mL of 15 N ammonia solution were added. The mixture was stirred at 100 °C for 18 hours. The resulting product **(2)** was isolated and purified, with a measured melting point of 280 °C. ^1H NMR (400 MHz, DMSO-d_6, Me_4Si) δ 8.85 (d, J = 3.2 Hz, 2H), 8.16 (s, 1H), 8.00 (d, J = 7.3 Hz, 1H), 7.90 (d, J = 3.2 Hz, 1H), 7.47 (d, J = 7.3 Hz, 1H), 3.89 (s, 3H, OMe).

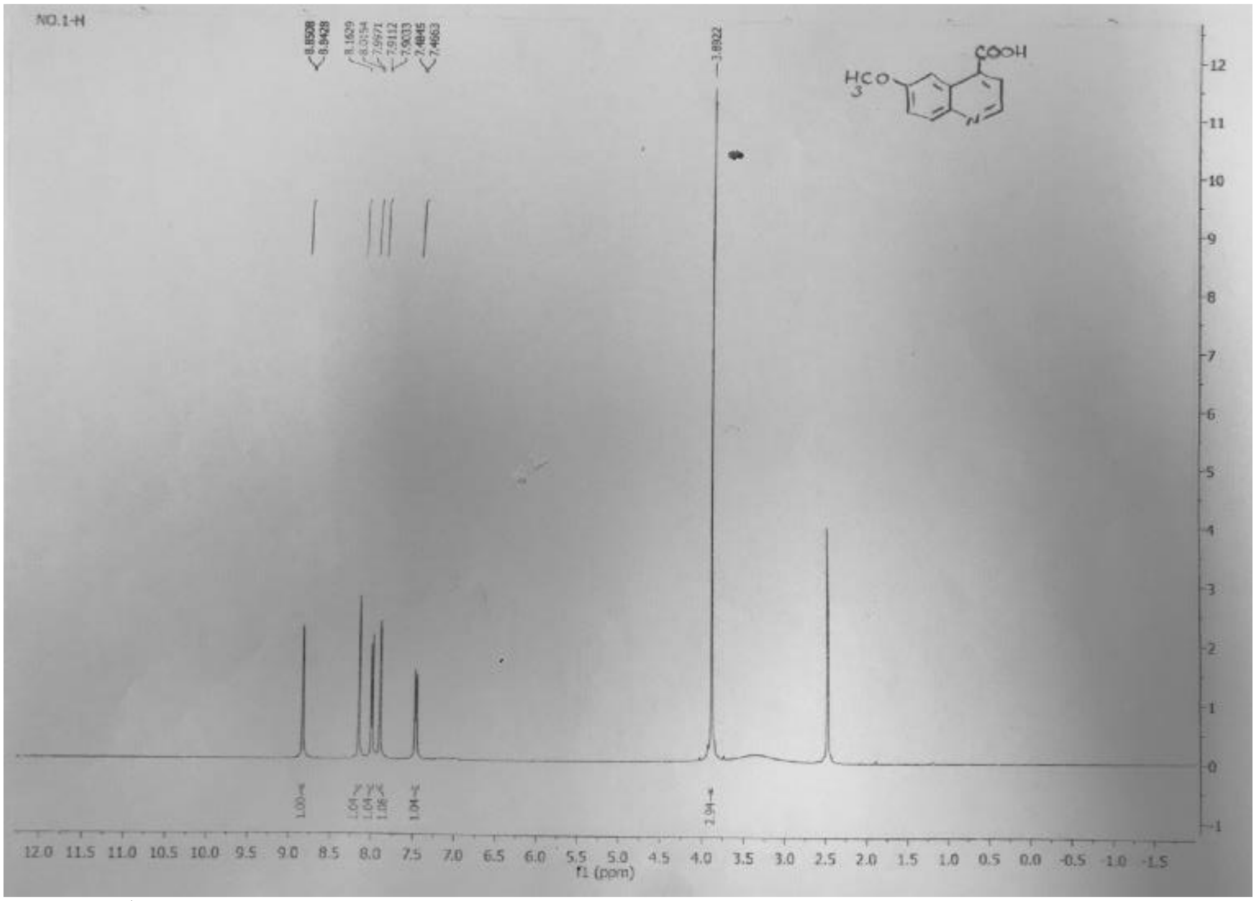

Figure S1. ^1H NMR spectrum of 4-carboxylic acid-6-methoxy quinoline (quinine acid) (**2**)

Synthesis of 6-hydroxyquinoline-4-carboxylic acid (3). The compound **(2)** (4.92 mmol) was added to 35 mL of 40% hydrobromic acid (HBr) solution. The reaction mixture was connected to a reflux condenser and heated for 30 hours. The product was isolated by standard work-up procedures, including filtration and washing with cold solvent. The resulting solid **(3)** was obtained with a melting point of 320 °C. ^1H NMR (400 MHz, DMSO-d_6, Me_4Si) δ 13.69 (brs, 1H, COOH),

10.29 (brs, 1H, OH), 8.78 (d, J = 3.2 Hz, 1H), 8.10 (s, 1H), 7.95 (d, J = 7.3 Hz, 1H), 7.85 (d, J = 3.1 Hz, 1H), 7.37 (d, J = 7.3 Hz, 1H).

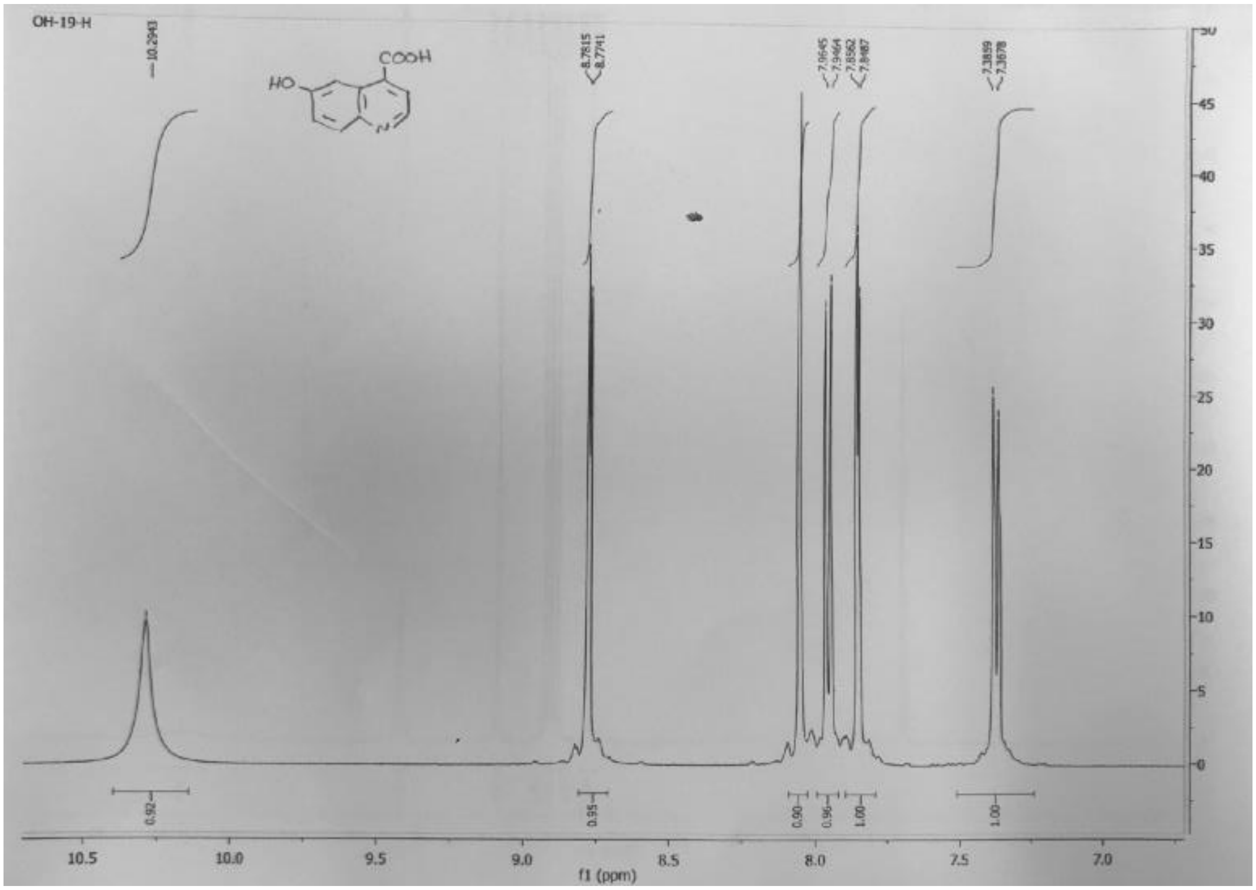

Figure S2. 1H NMR spectrum of 6-hydroxyquinoline-4-carboxylic acid (**3**)

Synthesis of ethyl 6-hydroxyquinoline-4-carboxylate (4). A solution of Compound **(3**) in absolute ethanol (20 ml) and concentrated H_2SO_4 (2 ml) was prepared and heated under reflux. After work-up, the product **(4)** was obtained as a solid with a melting point of 189 °C. ^{1}H NMR (400 MHz, DMSO-d_6, Me_4Si) δ 10.29 (brs, 1H, OH), 8.70 (d, J = 3.5 Hz, 1H), 7.96 (d, J = 7.8 Hz, 2H), 7.85 (d, J = 3.5 Hz, 1H), 7.39 (dd, J = 7.3, 1.9 Hz, 1H), 4.43 (q, J = 5.7 Hz, CH2), 1.39 (t, J = 5.7 Hz, CH3).

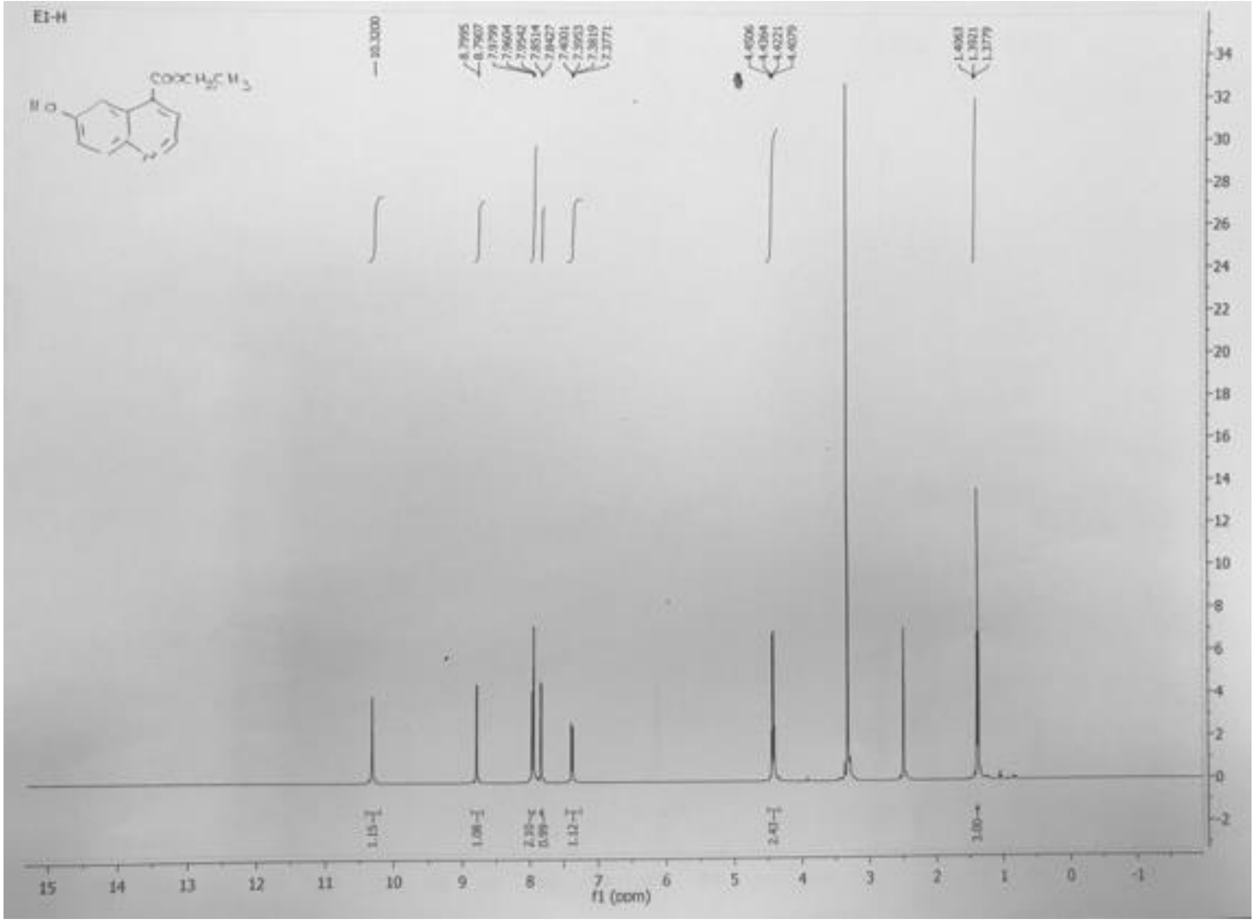

Figure S3. ^{1}H NMR spectrum of ethyl 6-hydroxyquinoline-4-carboxylate (**4**)

Synthesis of ethyl 6-(3-bromopropoxy)quinoline-4-carboxylate (5). Compound **(4)** (1mmol) was dissolved in 10 ml of acetonitrile, followed by the addition of potassium carbonate (K_2CO_3, 2mmol). Subsequently, 1,3-dibromopropane (12mmol) was added to the reaction flask, and the mixture was heated under reflux conditions. The product **(5)** was obtained as a solid. MS (m/z): Calculated for $C_{15}H_{16}BrNO_3$, exact Mass = 337.03; observed peak at m/z = 339.2. The mass spectrum exhibited two peaks of equal intensity at m/z 339.2 and 341.2, consistent with the characteristic isotopic pattern of bromine (Br-79 and Br-81). Additionally, a fragment ion was observed at m/z 257.1, which is attributed to the loss of the bromine atom (Br), further supporting the structure of the brominated compound. ^{1}H NMR (400 MHz, DMSO-d_6, Me_4Si) δ 8.88 (d, J = 3.5 Hz, 1H), 8.07 (d, J = 7.5 Hz, 1H), 8.05 (d, J = 7.5 Hz, 1H), 7.93 (d, J = 3.5 Hz, 1H), 7.51 (dd, J = 7.5, 1.8 Hz, 1H), 4.45 (q, J = 7.1 Hz, 2H, OCH_2CH_3), 4.22 (t, J = 6.8 Hz, 2H, CH_2Br), 3.75 (t, J = 6.8 Hz, 2H, CH_2O), 2.34 (dd, J = 7.1, 3.9 Hz, , 2H, -CH2-), 1.42 (t, J = 7.1 Hz, 3H, OCH_2CH_3).

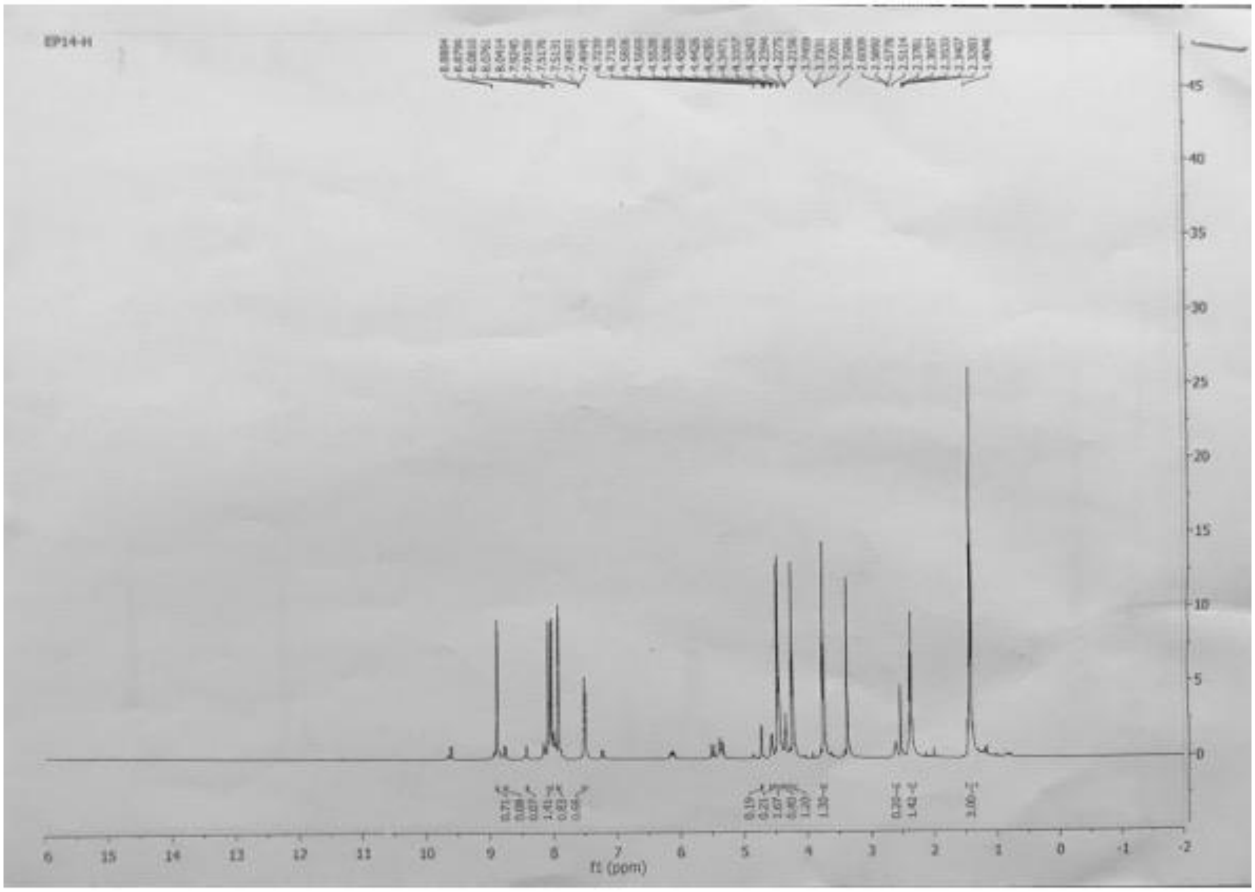

Figure S4. ¹H NMR spectrum of ethyl 6-(3-bromopropoxy)quinoline-4-carboxylate (**5**)

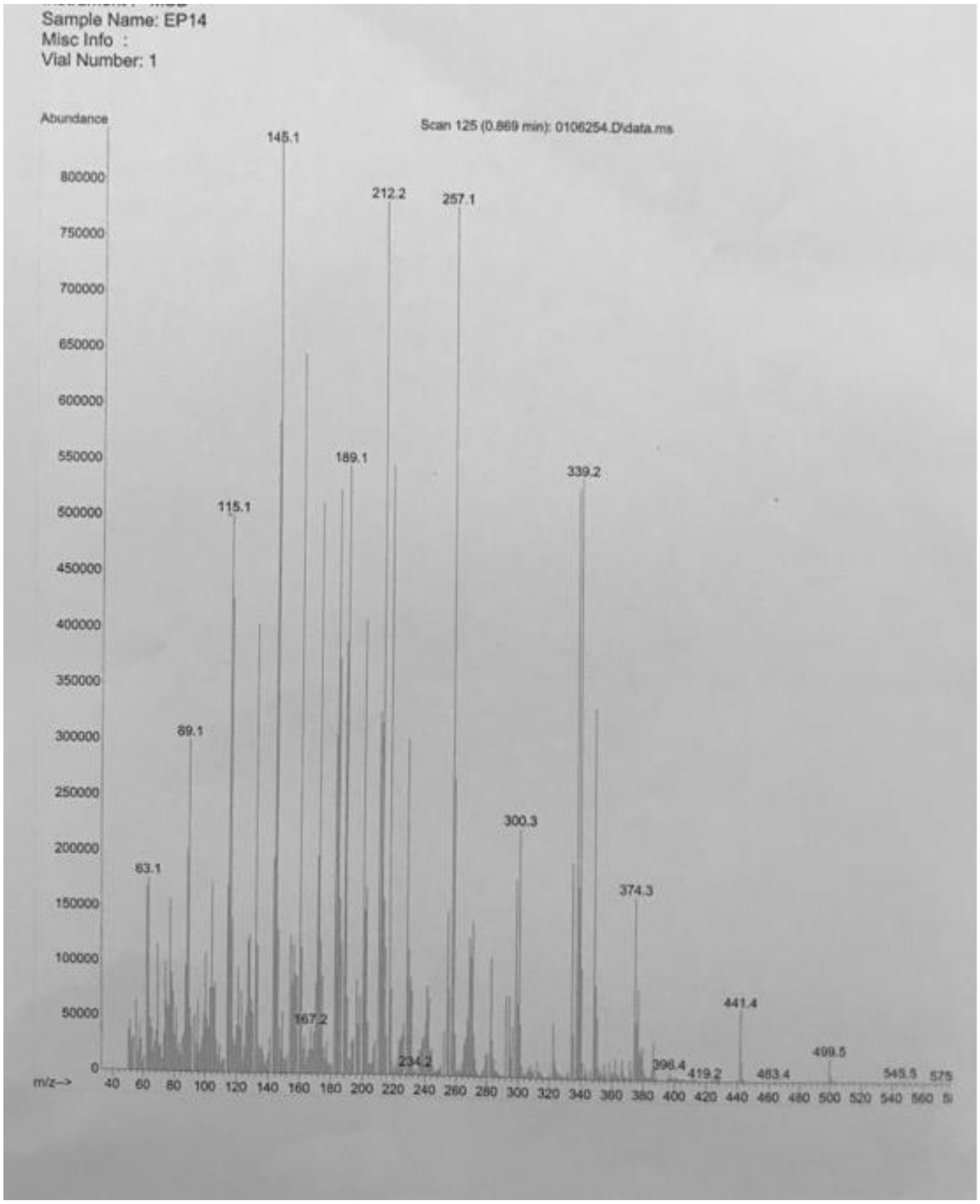

Figure S5. EI-MS spectrum of ethyl 6-(3-bromopropoxy)quinoline-4-carboxylate (**5**)

Synthesis of ethyl 6-(3-(4-(tert-butoxycarbonyl) piperazin-1-yl) propoxy)quinoline-4-carboxylate (6). A solution of compound **(5)** (1mmol), N-Boc piperazine (1mmol), potassium carbonate (K_2CO_3, 4mmol), and potassium iodide (KI, 0.5mmol) in acetone was stirred at room temperature. The purity of the product **(6)** was determined to be 99.6% by analytical HPLC, with a retention time (R_t) of 31.51 min. HRMS (ESI) (m/z): Calculated $[M + H]^+$ for $C_{24}H_{34}N_3O_5$ = 444.0721; found = 444.0716. ^{1}H NMR (400 MHz, DMSO-d_6, Me_4Si) δ 8.89 (d, J = 3.5 Hz, 1H), 8.09 (d, J = 7.3 Hz, 1H), 8.05 (d, J = 7.5 Hz, 1H), 7.94 (d, J = 3.5 Hz, 1H), 7.50 (dd, J = 7.5, 1.9 Hz, 1H), 4.48 (q, J = 6.9 Hz, 2H, OCH_2CH_2), 4.18 (t, J = 7.1 Hz, 2H, OCH_2CH_3), 3.49 (m, 2H, CH_2 of piperazine), 2.50 (m, 4H, CH_2CH_2 of piperazine and propoxy group), 1.12 (t, J = 7.1 Hz, 3H, OCH_2CH_3), 1.07 (s, 9H, $C(CH_3)_3$).

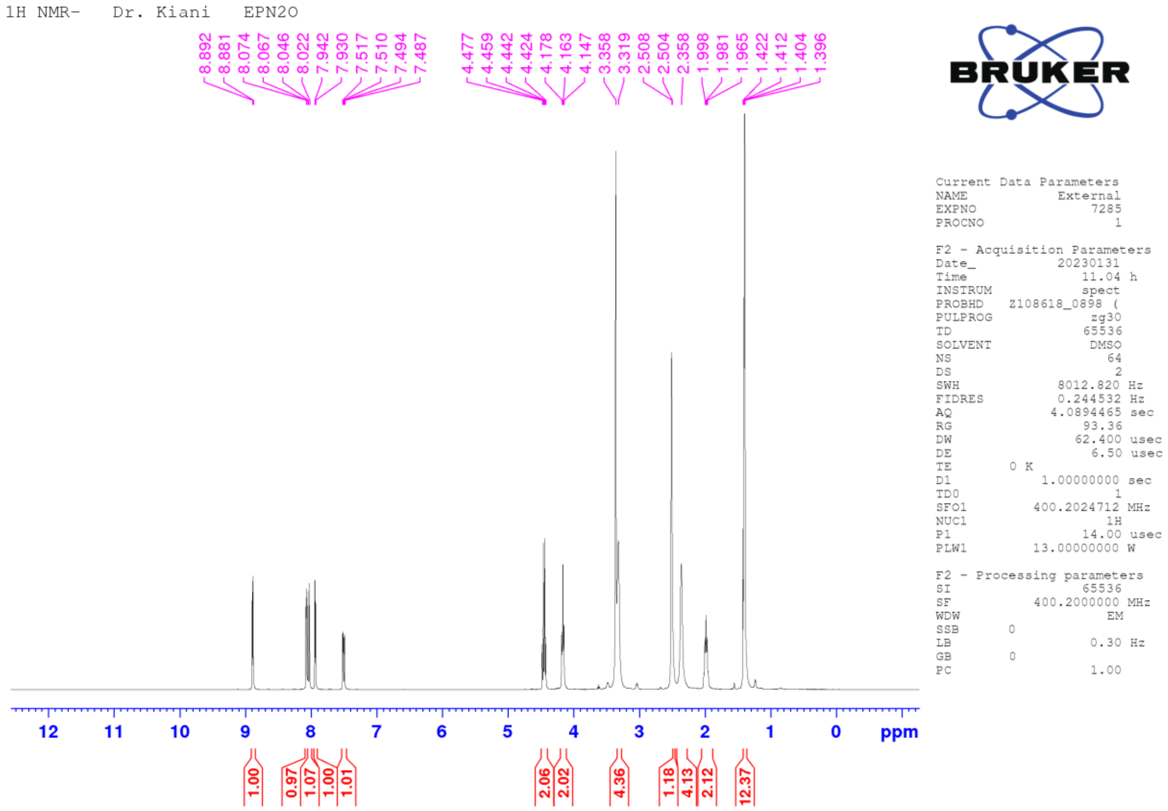

Figure S6. ^{1}H NMR spectrum of ethyl 6-(3-(4-(tert-butoxycarbonyl) piperazin-1-yl) propoxy)quinoline-4-carboxylate (**6**)

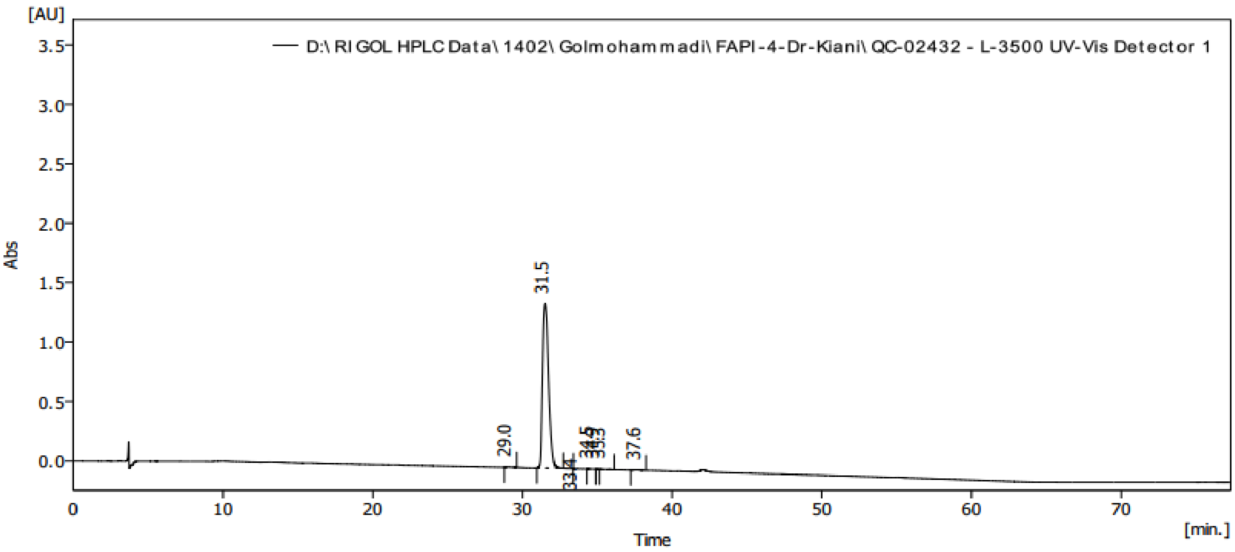

Result Table (Uncal - D:\RIGOL HPLC Data\1402\Golmohammadi\FAPI-4-Dr-Kiani\QC-02432 - L-3500 UV-Vis Detector 1)

	Reten. Time [min]	Area [mAU.s]	Height [mAU]	Area [%]	Height [%]	W05 [min]	Compound Name
1	28.997	61.443	2.676	0.2	0.2	0.28	
2	31.513	40202.553	1382.585	99.6	99.5	0.46	
3	33.400	0.023	0.007	0.0	0.0	0.01	
4	34.470	29.013	1.172	0.1	0.1	0.27	
5	34.903	3.911	0.411	0.0	0.0	0.04	
6	35.267	30.773	0.822	0.1	0.1	0.25	
7	37.633	48.509	2.109	0.1	0.2	0.32	
	Total	40376.225	1389.783	100.0	100.0		

Figure S7. Analytical HPLC chromatogram of ethyl 6-(3-(4-(tert-butoxycarbonyl) piperazin-1-yl) propoxy)quinoline-4-carboxylate (**6**)

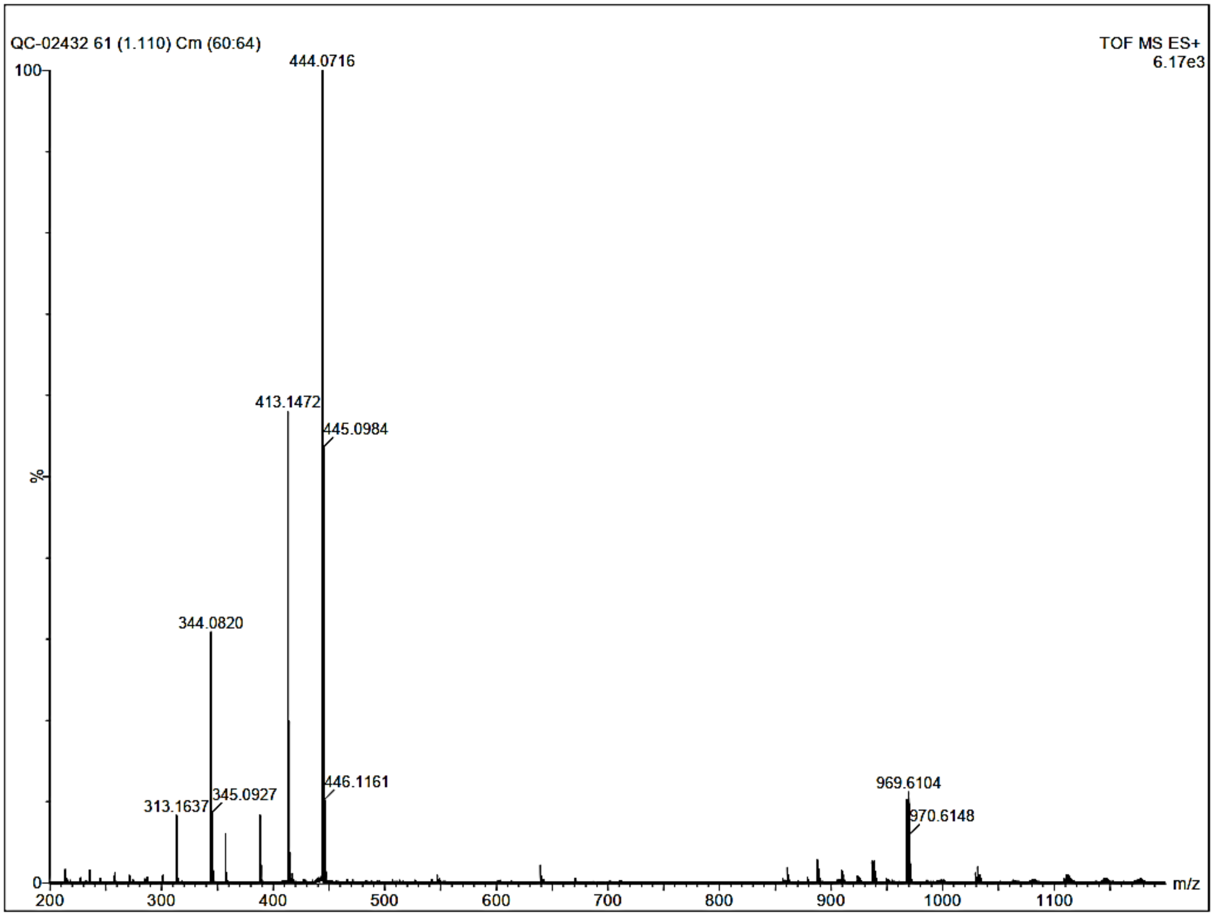

Figure S8. HRMS-ESI spectra of ethyl 6-(3-(4-(tert-butoxycarbonyl) piperazin-1-yl) propoxy)quinoline-4-carboxylate (**6**)

Synthesis of 6-(3-(4-(tert-butoxycarbonyl) piperazin-1-yl) propoxy) quinoline-4-carboxylic acid (7). Compound **(6)** (1.2 mmol, 532 mg) was dissolved in a mixture of tetrahydrofuran (THF, 4.8 ml) and distilled water (7.2 ml). The reaction mixture was stirred at room temperature for 10 minutes. Lithium hydroxide (LiOH, 1.8 mmol, 43.11 mg) was added, and stirring was continued until complete consumption of the starting material. The product was purified by preparative HPLC and isolated by freeze-drying to yield compound **(7)** (480 mg, 96%) with a purity of more than 99.3% (R_t: 20.27 min). HRMS (ESI) m/z: Calculated for $C_{22}H_{30}N_3O_5$ $[M + H]^+$ 416.0138, Found 416.0131, Calculated for $C_{44}H_{59}N_6O_{10}$ $[2M + H]^+$ 831.5759, Found 831.5753.

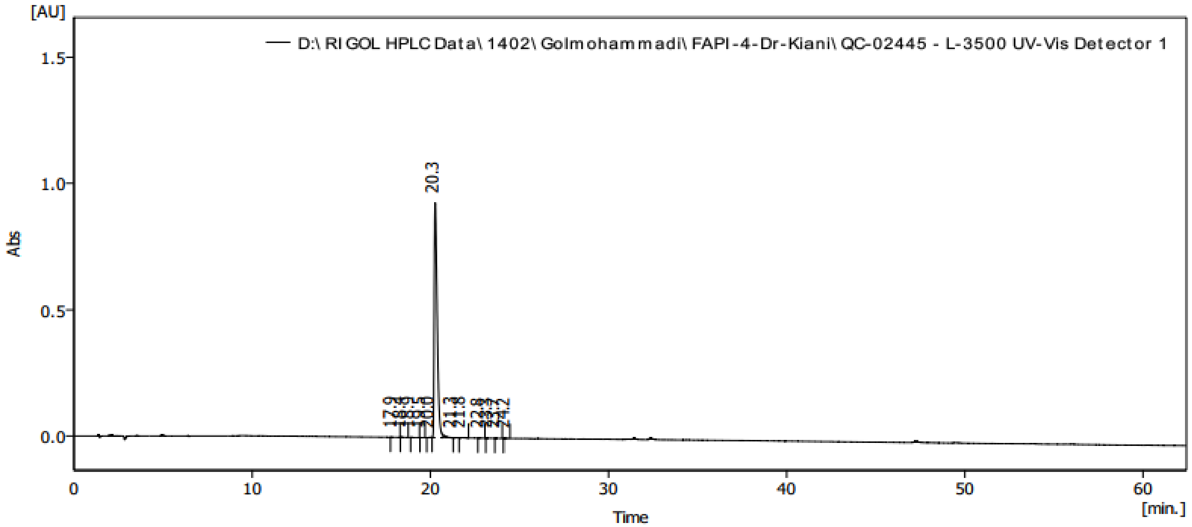

Result Table (Uncal - D:\RIGOL HPLC Data\1402\Golmohammadi\FAPI-4-Dr-Kiani\QC-02445 - L-3500 UV-Vis Detector 1)

	Reten. Time [min]	Area [mAU.s]	Height [mAU]	Area [%]	Height [%]	W05 [min]	Compound Name
1	17.900	3.588	0.189	0.0	0.0	0.26	
2	18.410	2.775	0.214	0.0	0.0	0.18	
3	18.897	2.054	0.024	0.0	0.0	0.02	
4	19.503	1.719	0.193	0.0	0.0	0.13	
5	19.993	13.659	1.359	0.1	0.1	0.19	
6	20.273	10827.849	931.366	99.3	99.4	0.18	
7	21.273	11.250	0.727	0.1	0.1	0.25	
8	21.757	17.456	1.296	0.2	0.1	0.20	
9	22.790	2.948	0.295	0.0	0.0	0.15	
10	23.283	6.377	0.393	0.1	0.0	0.22	
11	23.710	3.958	0.192	0.0	0.0	0.26	
12	24.240	6.995	0.743	0.1	0.1	0.15	
	Total	10900.628	936.990	100.0	100.0		

Figure S9. Analytical HPLC chromatogram of 6-(3-(4-(tert-butoxycarbonyl) piperazin-1-yl) propoxy) quinoline-4-carboxylic acid (**7**)

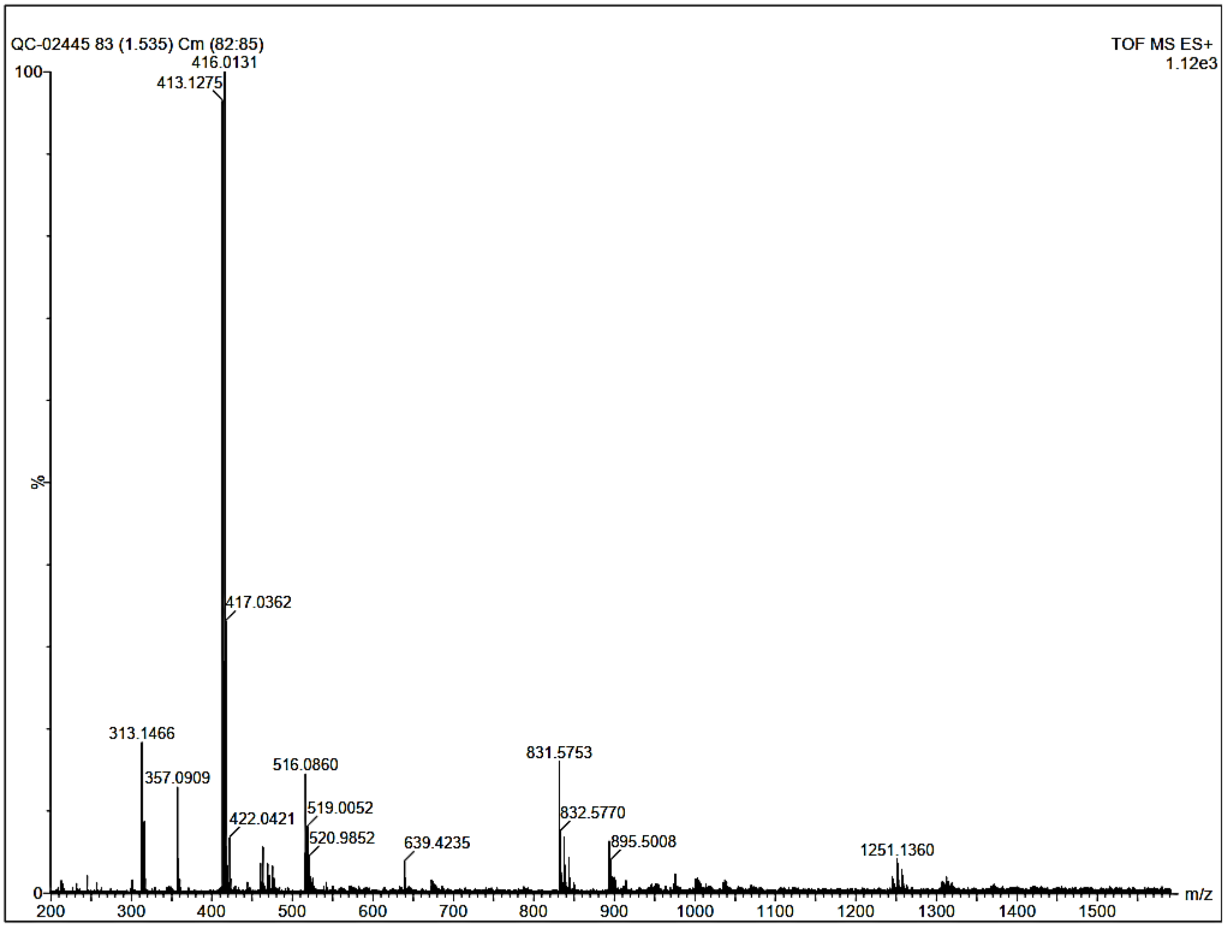

Figure S10. HRMS-ESI spectra of 6-(3-(4-(tert-butoxycarbonyl) piperazin-1-yl) propoxy) quinoline-4-carboxylic acid (7)

Synthesis of tert-butyl (S)-4-(3-((4-((2-(2-cyano-4,4-difluoropyrrolidin-1-yl)-2-oxoethyl) carbamoyl) quinolin-6-yl) oxy) propyl) piperazine-1-carboxylate (8). To a solution of compound **(7)** (1.0 equivalent, 1.11 mmol, 461.2 mg) in DMF (8 ml), some reagents were added, including TBTU (1.25 equivalent, 1.39 mmol, 447 mg), HOBt (2.80 equivalent, 3.11 mmol, 420 mg), and DIPEA (3.0 equivalent, 3.33 mmol, 566 µl). The reaction mixture was stirred at room temperature for 15 min. Subsequently, a solution of (*S*)-1-(2-aminoacetyl) pyrrolidine-2-carbonitrile 4-methylbenzenesulfonate (1.33 mmol, 1.2 equivalent, 252 mg) in DMF (2ml) was added. The mixture was quenched with water (20 ml) and purified by preparative HPLC. Freeze drying provided the compound **(8)** (470 mg, 75% yield) with a purity of more than 98.9%(R_t: 22.87 min). HRMS (ESI) m/z: Calculated for $C_{29}H_{37}F_2N_6O_5$ $[M + H]^+$ 587.1506, Found 587.1500.

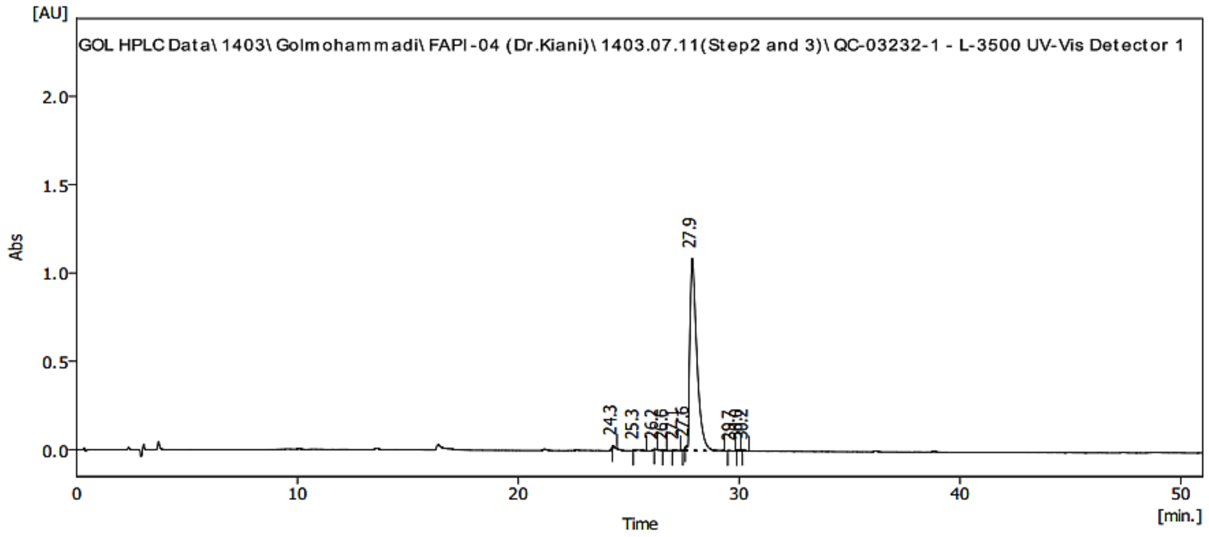

Result Table (Uncal - D: \RIGOL HPLC Data\1403\Golmohammadi\FAPI-04 (Dr.Kiani)\1403.07.11(Step2 and 3)\QC-03232-1 - L-3500 UV-Vis Detector 1)

	Reten. Time [min]	Area [mAU.s]	Height [mAU]	Area [%]	Height [%]	W05 [min]	Compound Name
1	24.297	63.314	10.366	0.3	0.9	0.09	
2	25.290	61.229	4.281	0.3	0.4	0.14	
3	26.177	5.988	1.161	0.0	0.1	0.05	
4	26.597	9.054	1.402	0.0	0.1	0.10	
5	27.100	25.303	2.473	0.1	0.2	0.16	
6	27.550	55.208	18.604	0.2	1.6	0.05	
7	27.877	23781.181	1087.530	98.9	96.1	0.34	
8	29.657	9.805	0.838	0.0	0.1	0.21	
9	29.990	20.406	3.097	0.1	0.3	0.12	
10	30.237	14.805	1.526	0.1	0.1	0.15	
	Total	24046.293	1131.280	100.0	100.0		

Figure S11. Analytical HPLC chromatogram of (S)-4-(3-((4-((2-(2-cyano-4,4-difluoropyrrolidin-1-yl)-2-oxoethyl) carbamoyl) quinolin-6-yl) oxy) propyl) piperazine-1-carboxylate (**8**)

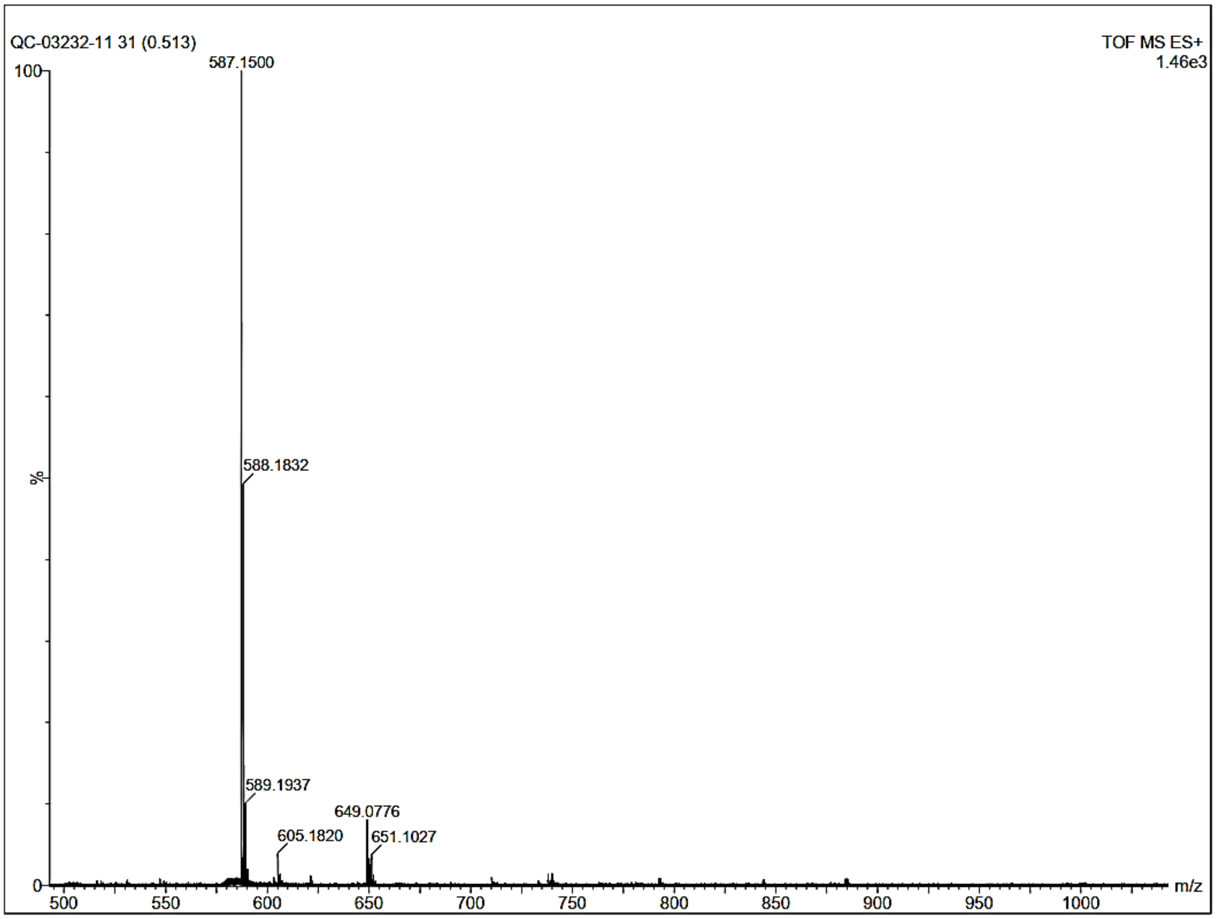

Figure S12. HRMS-ESI sepectra of (S)-4-(3-((4-((2-(2-cyano-4,4-difluoropyrrolidin-1-yl)-2-oxoethyl) carbamoyl) quinolin-6-yl) oxy) propyl) piperazine-1-carboxylate (**8**)

Synthesis of (9H-fluoren-9-yl) methyl (S)-(15-(4-(3-((4-((2-(2-cyano-4,4-difluoropyrrolidin-1-yl)-2-oxoethyl) carbamoyl) quinolin-6-yl) oxy) propyl) piperazin-1-yl)-15-oxo-3,6,9,12-tetraoxapentadecyl) carbamate (9). Compound **(8)** (452 mg, 0.77 mmol) was dissolved in a mixture of acetonitrile (5 ml) and trifluoroacetic acid (15 ml). The reaction mixture was shaken for 15 minutes at room temperature, and the residue was precipitated with diethyl ether. The resulting solid was collected by centrifugation and dissolved in *N*, *N*-dimethylformamide(DMF, 10 mL). To the resulting solution, some reagent was added, including DIPEA (5 mL), TBTU (2.20 equivalents, 1.7 mmol, 546 mg), and Fmoc-NH(PEG)$_3$-COOH (2.20 equivalents, 1.7 mmol, 829 mg). The reaction mixture was stirred at room temperature for 5 hours. After completion (monitored by TLC), the mixture was diluted with water (20 mL) and purified by preparative HPLC. The compound **(9)** (478 mg, 65% yield) was obtained with a purity of more than 98.6% (R_t: 38.39 min in HPLC analysis). HRMS (ESI) m/z: Calculated for $C_{50}H_{60}F_2N_7O_{10}$ $[M + H]^+$ 956.1440, Found 956.1436.

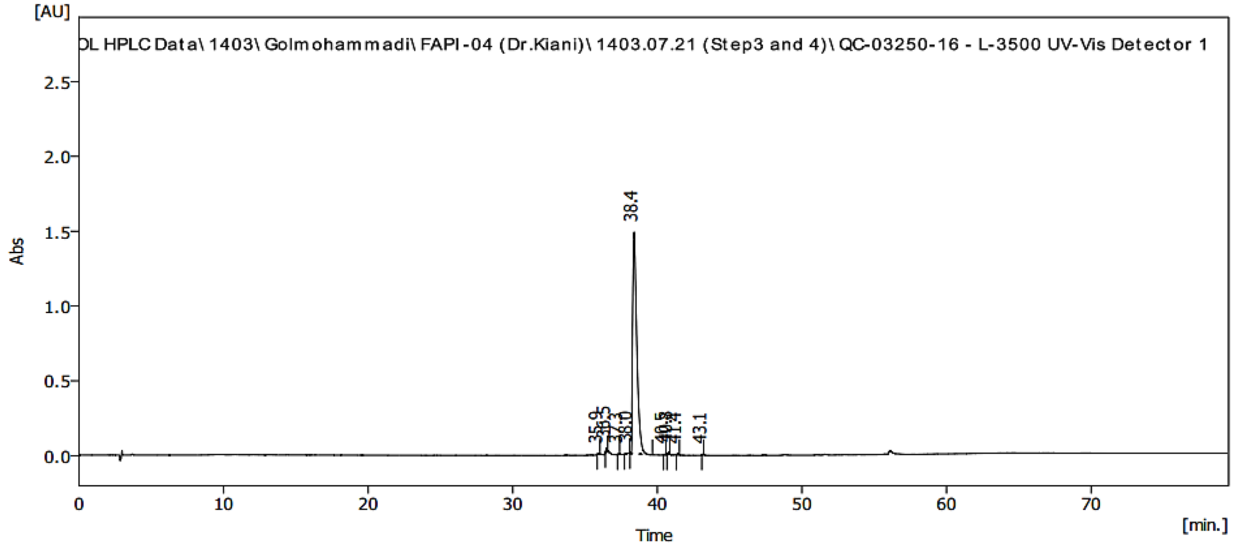

Result Table (Uncal - D:\RIGOL HPLC Data\1403\Golmohammadi\FAPI-04 (Dr.Kiani)\1403.07.21 (Step3 and 4)\QC-03250-16 - L-3500 UV-Vis Detector 1)

	Reten. Time [min]	Area [mAU.s]	Height [mAU]	Area [%]	Height [%]	W05 [min]	Compound Name
1	35.947	29.923	3.501	0.1	0.2	0.15	
2	36.480	133.593	22.702	0.4	1.5	0.11	
3	37.340	15.436	2.839	0.1	0.2	0.09	
4	37.997	41.354	2.872	0.1	0.2	0.13	
5	38.390	29528.434	1481.595	98.6	96.1	0.31	
6	40.507	22.547	3.906	0.1	0.3	0.10	
7	40.773	82.170	12.638	0.3	0.8	0.11	
8	41.413	58.543	8.031	0.2	0.5	0.12	
9	43.143	21.072	4.076	0.1	0.3	0.09	
	Total	29933.072	1542.159	100.0	100.0		

Figure S13. Analytical HPLC chromatogram of (9H-fluoren-9-yl) methyl (S)-(15-(4-(3-((4-((2-(2-cyano-4,4-difluoropyrrolidin-1-yl)-2-oxoethyl) carbamoyl) quinolin-6-yl) oxy) propyl) piperazin-1-yl)-15-oxo-3,6,9,12-tetraoxapentadecyl) carbamate (**9**)

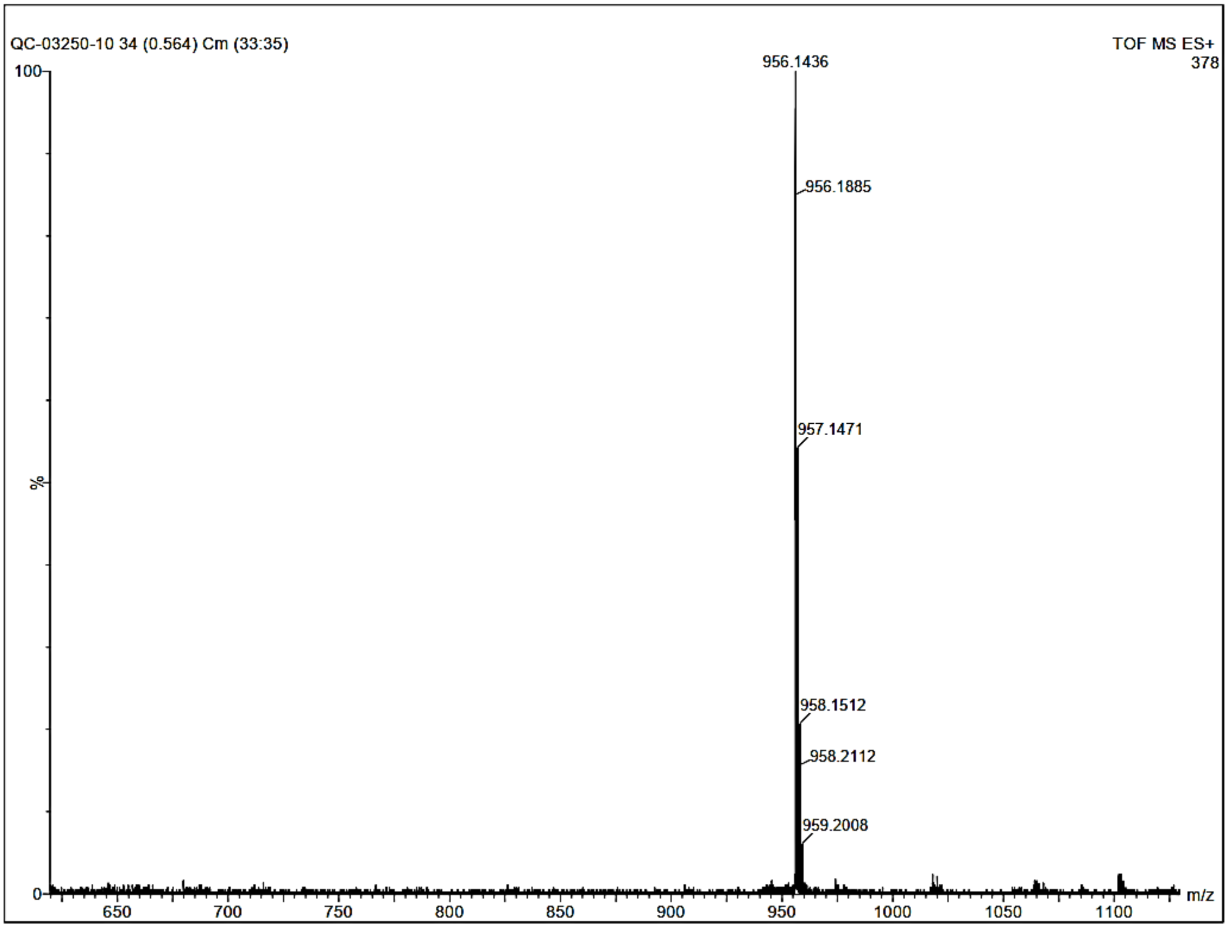

Figure S14. HRMS-ESI spectra of (9H-fluoren-9-yl) methyl (S)-(15-(4-(3-((4-((2-(2-cyano-4,4-difluoropyrrolidin-1-yl)-2-oxoethyl) carbamoyl) quinolin-6-yl) oxy) propyl) piperazin-1-yl)-15-oxo-3,6,9,12-tetraoxapentadecyl) carbamate (**9**)

Synthesis of (S)-6-(3-(4-(1-amino-3,6,9,12-tetraoxapentadecan-15-oyl) piperazin-1-yl) propoxy)-N-(2-(2-cyano-4,4-difluoropyrrolidin-1-yl)-2-oxoethyl)quinoline-4-carboxamide (10). Compound **(9)** (0.49 mmol, 468 mg) was dissolved in a solution of 20% (v/v) piperidine in ethyl acetate (15 mL) in a round-bottom flask. The reaction mixture was stirred at room temperature for 3 hours, and the progress of the reaction was monitored by using analytical HPLC. After completion, the reaction mixture was purified by preparative HPLC. The compound **(10)** (198 mg, 55%, yield) was obtained with a purity of more than 99.10 % (R_t: 19.30 min). HRMS (ESI) m/z: Calculated for $C_{35}H_{50}F_2N_7O_8$ $[M + H]^+$ 734.2432, Found 734.2438.

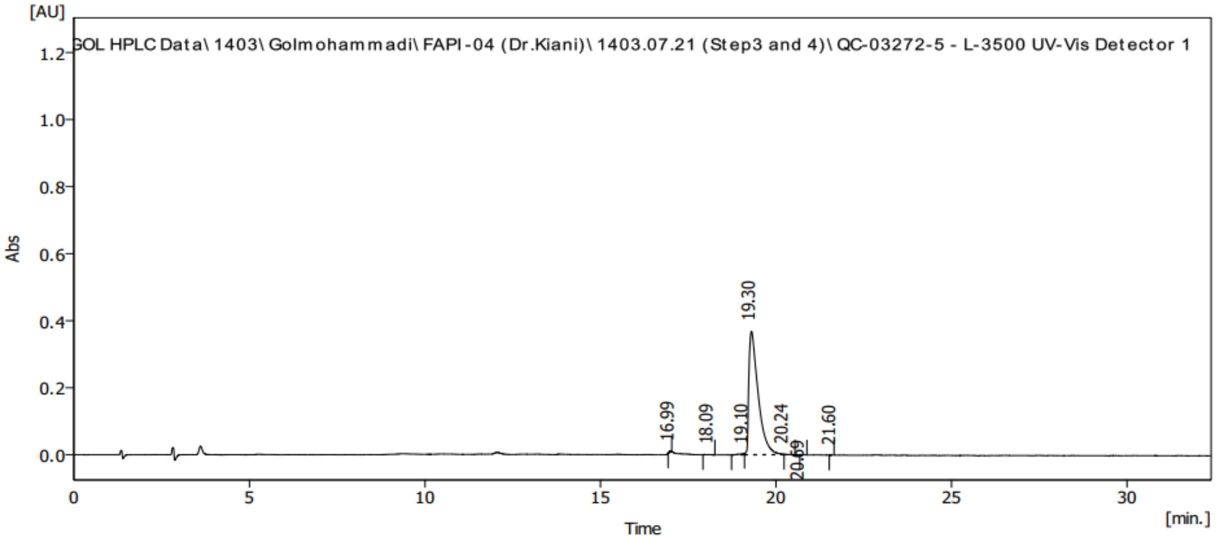

Result Table (Uncal - D:\RIGOL HPLC Data\1403\Golmohammadi\FAPI-04 (Dr.Kiani)\1403.07.21 (Step3 and 4)\QC-03272-5 - L-3500 UV-Vis Detector 1)

	Reten. Time [min]	Area [mAU.s]	Height [mAU]	Area [%]	Height [%]	W05 [min]	Compound Name
1	16.990	13.902	3.285	0.2	0.9	0.07	
2	18.093	3.481	0.409	0.1	0.1	0.14	
3	19.097	35.739	3.823	0.5	1.0	0.16	
4	19.300	6778.008	368.652	99.1	97.7	0.28	
5	20.237	9.483	0.909	0.1	0.2	0.18	
6	20.690	0.965	0.031	0.0	0.0	0.02	
7	21.597	1.167	0.255	0.0	0.1	0.08	
	Total	6842.744	377.364	100.0	100.0		

Figure S15. Analytical HPLC chromatogram of (S)-6-(3-(4-(1-amino-3,6,9,12-tetraoxapentadecan-15-oyl) piperazin-1-yl) propoxy)-N-(2-(2-cyano-4,4-difluoropyrrolidin-1-yl)-2-oxoethyl) quinoline-4-carboxamide (**10**)

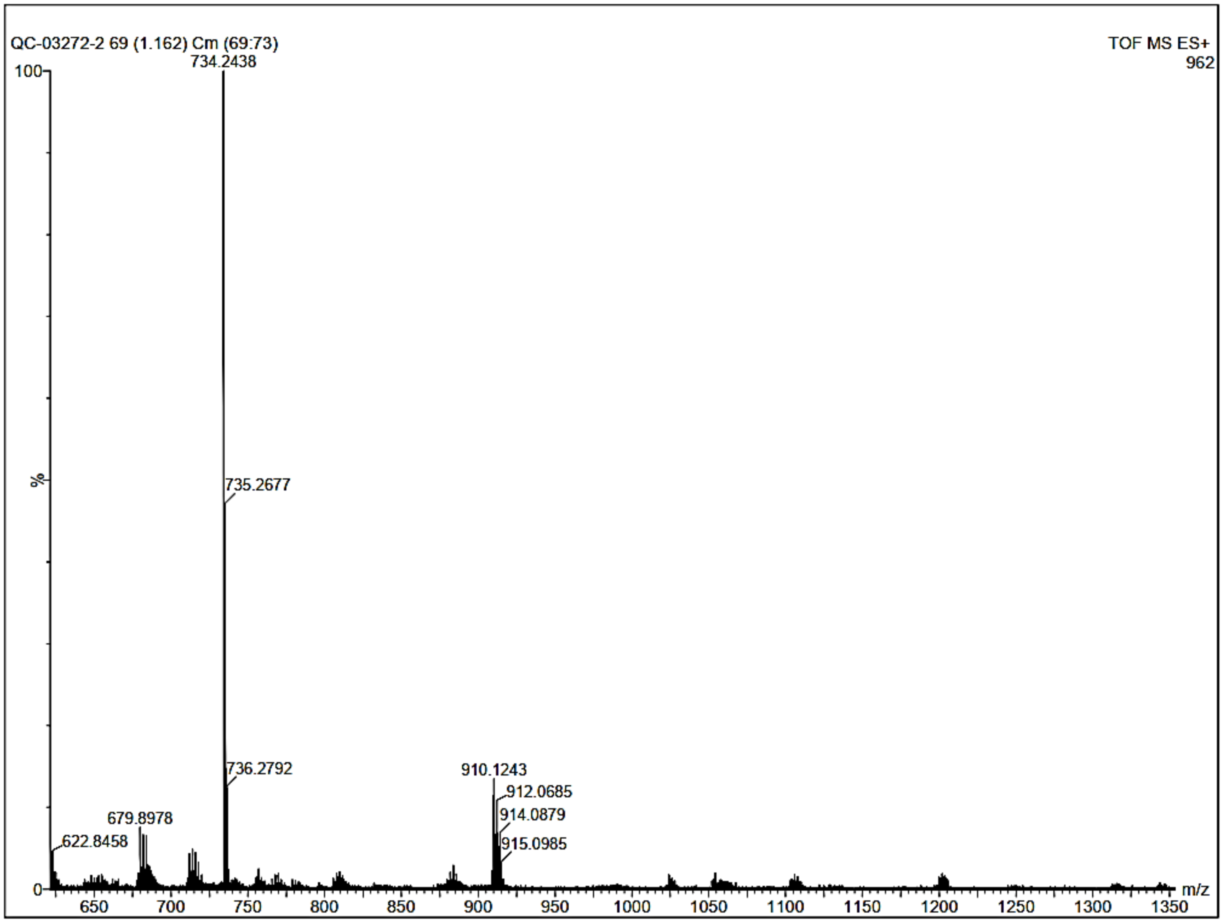

Figure S16. HRMS-ESI spectra of (S)-6-(3-(4-(1-amino-3,6,9,12-tetraoxapentadecan-15-oyl) piperazin-1-yl) propoxy)-N-(2-(2-cyano-4,4-difluoropyrrolidin-1-yl)-2-oxoethyl) quinoline-4-carboxamide (**10**)

Synthesis of tri-tert-butyl 2,2',2''-(10-(18-(4-(3-((4-((2-(2-cyano-4,4-difluoropyrrolidin-1-yl)-2-oxoethyl) carbamoyl) quinolin-6-yl) oxy) propyl) piperazin-1-yl)-2,18-dioxo-6,9,12,15-tetraoxa-3-azaoctadecyl)-1,4,7,10-tetraazacyclododecane-1,4,7-triyl) (R)-triacetate (11). A solution of TBTU (0.2706 mmol, 87 mg), DOTA-tris (*tert*-butyl ester) (0.2706 mmol, 155 mg), and DIPEA (0.369 mmol, 63 µl) was prepared in DMF (3ml), and the mixture was stirred at room temperature for 15 minutes. Then compound **(10)** (1.0 equivalent, 0.123 mmol, 90 mg) was added, and the reaction mixture and stirred at room temperature for 5 hours. The progress of the reaction was monitored using analytical HPLC. After completion, the reaction mixture was diluted with water (10 ml) and purified by preparative HPLC. The compound **(11)** (111 mg, 70% yield) was obtained with a purity of more than 99.10% (R_t: 35.04 min). HRMS (ESI) m/z: Calculated for $C_{63}H_{100}F_2N_{11}O_{15}$ $[M + H]^+$ 1288.3772, Found 1288.3767.

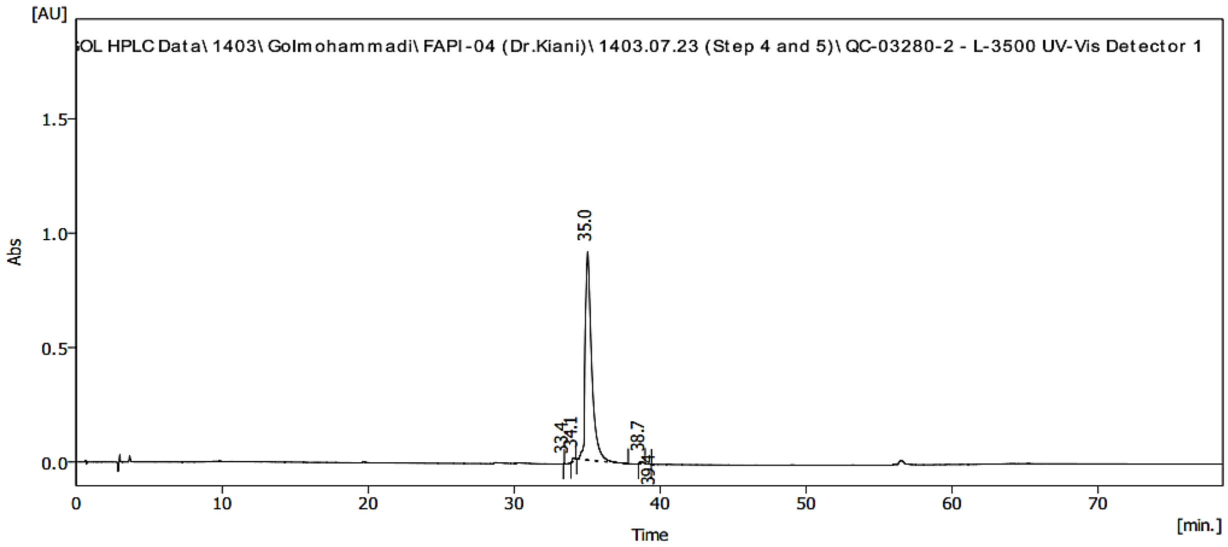

Result Table (Uncal - D:\RIGOL HPLC Data\1403\Golmohammadi\FAPI-04 (Dr.Kiani)\1403.07.23 (Step 4 and 5)\QC-03280-2 - L-3500 UV-Vis Detector 1)

	Reten. Time [min]	Area [mAU.s]	Height [mAU]	Area [%]	Height [%]	W05 [min]	Compound Name
1	33.433	0.130	0.034	0.0	0.0	0.01	
2	34.070	149.415	14.491	0.5	1.6	0.23	
3	35.040	27805.218	910.584	99.1	97.8	0.45	
4	38.663	110.127	6.311	0.4	0.7	0.32	
5	39.397	0.010	0.013	0.0	0.0	0.01	
	Total	28064.899	931.434	100.0	100.0		

Figure S17. Analytical HPLC chromatogram of tri-tert-butyl 2,2',2''-(10-(18-(4-(3-((4-((2-(2-cyano-4,4-difluoropyrrolidin-1-yl)-2-oxoethyl) carbamoyl) quinolin-6-yl) oxy) propyl) piperazin-1-yl)-2,18-dioxo-6,9,12,15-tetraoxa-3-azaoctadecyl)-1,4,7,10-tetraazacyclododecane-1,4,7-triyl) (R)-triacetate (**11**)

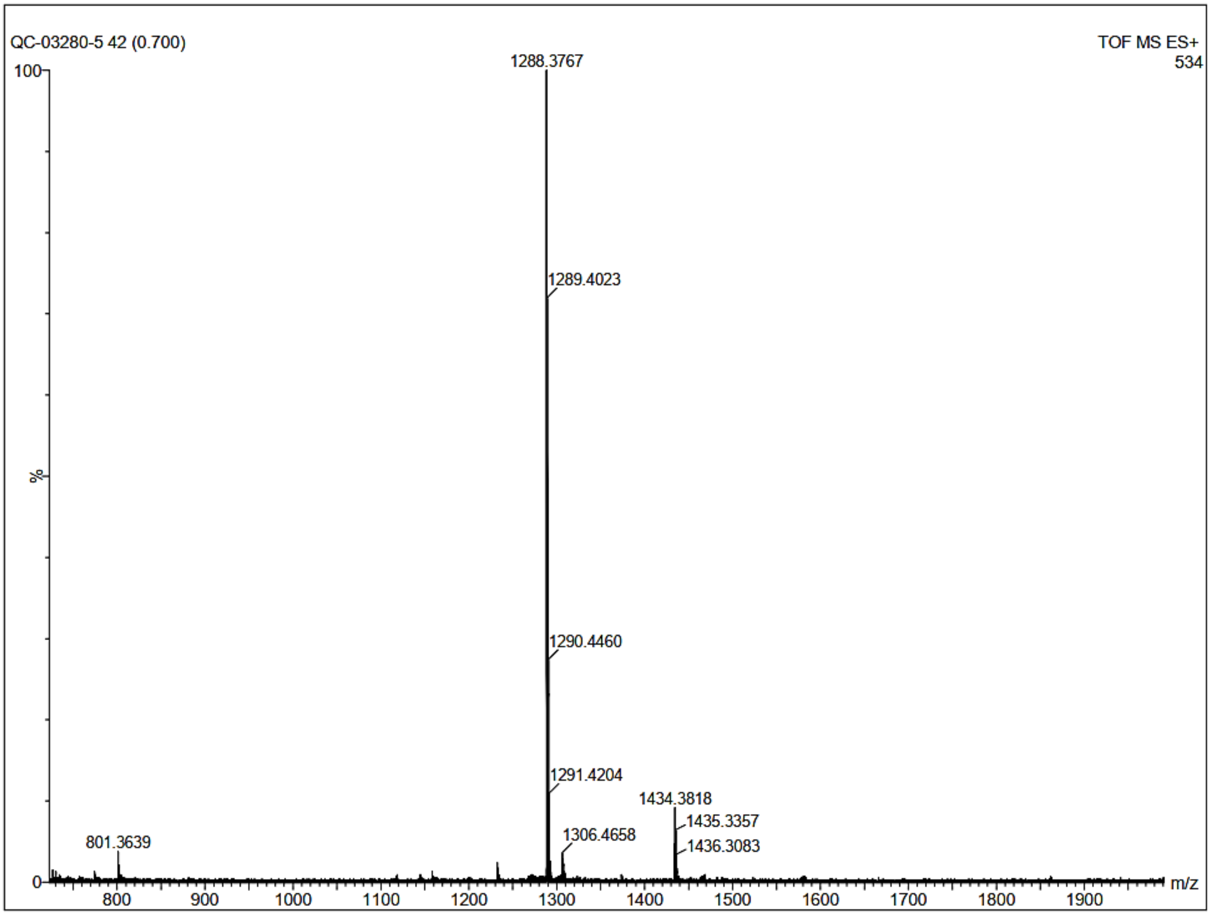

Figure S18 HRMS-ESI spectra of tri-tert-butyl 2,2',2"-(10-(18-(4-(3-((4-((2-(2-cyano-4,4-difluoropyrrolidin-1-yl)-2-oxoethyl) carbamoyl) quinolin-6-yl) oxy) propyl) piperazin-1-yl)-2,18-dioxo-6,9,12,15-tetraoxa-3-azaoctadecyl)-1,4,7,10-tetraazacyclododecane-1,4,7-triyl) (R)-triacetate (**11**)

Synthesis of (R)-2,2',2''-(10-(18-(4-(3-((4-((2-(2-cyano-4,4-difluoropyrrolidin-1-yl)-2-oxoethyl) carbamoyl) quinolin-6-yl) oxy) propyl) piperazin-1-yl)-2,18-dioxo-6,9,12,15-tetraoxa-3-azaoctadecyl)-1,4,7,10-tetraazacyclododecane-1,4,7-triyl) triacetic acid (FAPI-MKG (12). Compound **(11)** (0.078 mmol, 100 mg) was treated with 10 ml of regent K {trifluoroacetic acid (TFA) / triethylsilane (TES) / H_2O / methanol (MeOH) (95%: 2.5%: 1.25%: 1.25%, v/v)} for 3 hours at room temperature to remove all the protecting groups. The mixture reaction was then precipitated in diethyl ether. Preparative HPLC purified the final product. Freeze drying provided the compound **(FAPI-MKG, 12)** (25 mg, 8% yield) as a white solid. HPLC analysis found that compound **(FAPI-MKG, 12)** was obtained in 98.10 %< purity (R_t: 21.08 min); HRMS (ESI) m/z: Calculated for $C_{51}H_{76}F_2N_{11}O_{15}$ $[M+H]^+$ 1120.2161, Found 1120.2166, Calculated for $C_{51}H_{77}F_2N_{11}O_{15}$ $[M + 2H]^{2+}$ 560.6311, Found 560.6307.

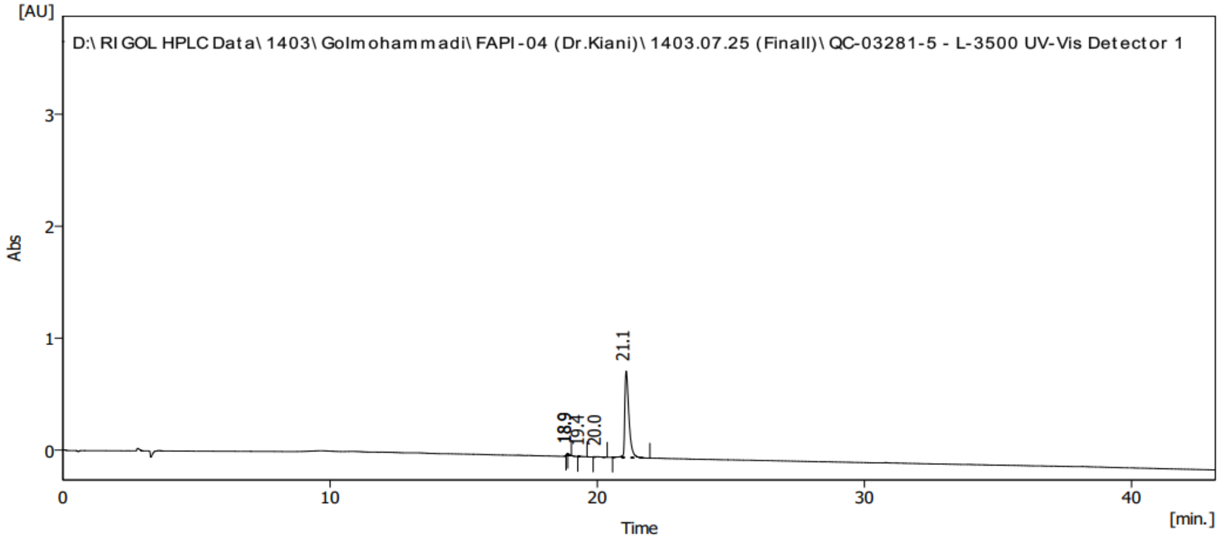

Result Table (Uncal - D:\RIGOL HPLC Data\1403\Golmohammadi\FAPI-04 (Dr.Kiani)\1403.07.25 (Finall)\QC-03281-5 - L-3500 UV-Vis Detector 1)

	Reten. Time [min]	Area [mAU.s]	Height [mAU]	Area [%]	Height [%]	W05 [min]	Compound Name
1	18.890	40.498	17.474	0.5	2.2	0.04	
2	18.900	66.127	17.258	0.8	2.1	0.06	
3	19.387	12.651	1.753	0.2	0.2	0.11	
4	20.013	39.763	3.550	0.5	0.4	0.14	
5	21.083	8251.081	770.909	98.1	95.1	0.16	
	Total	8410.121	810.943	100.0	100.0		

Figure S19. Analytical HPLC chromatogram of (R)-2,2',2''-(10-(18-(4-(3-((4-((2-(2-cyano-4,4-difluoropyrrolidin-1-yl)-2-oxoethyl) carbamoyl) quinolin-6-yl) oxy) propyl) piperazin-1-yl)-2,18-dioxo-6,9,12,15-tetraoxa-3-azaoctadecyl)-1,4,7,10-tetraazacyclododecane-1,4,7-triyl) triacetic acid (**12**)

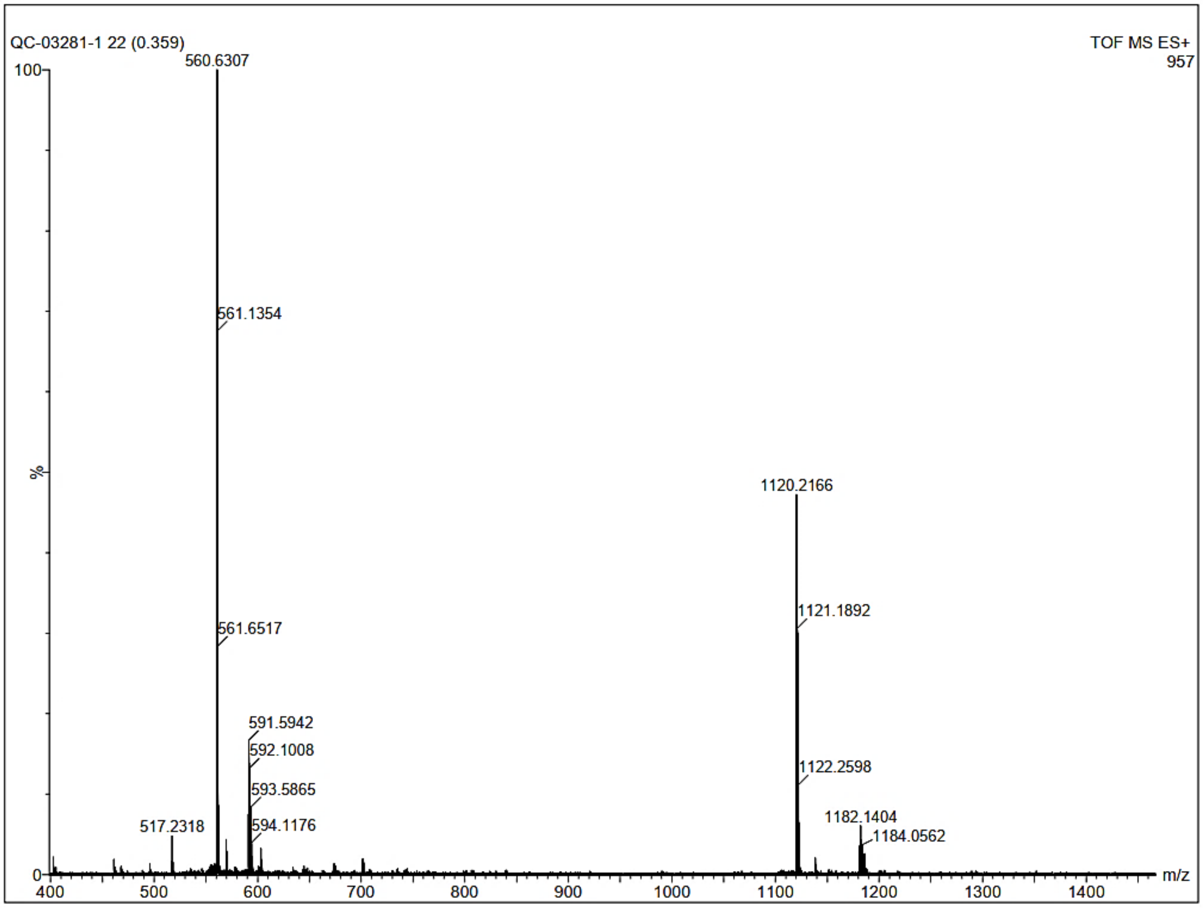

Figure S20. HRMS-ESI spectra of (R)-2,2',2''-(10-(18-(4-(3-((4-((2-(2-cyano-4,4-difluoropyrrolidin-1-yl)-2-oxoethyl) carbamoyl) quinolin-6-yl) oxy) propyl) piperazin-1-yl)-2,18-dioxo-6,9,12,15-tetraoxa-3-azaoctadecyl)-1,4,7,10-tetraazacyclododecane-1,4,7-triyl) triacetic acid (**12**)

S2. Molecular Docking

S2.1. Preparation of Structures FAP and Ligands

The crystal structure of FAP was obtained from the Protein Data Bank (PDB ID: 1Z68). Briefly, some processes were performed on the 3D structures, including the deletion of water molecules and non-essential N-acetylglucosamine (NAG) residues using UCSF Chimera software, assigning bond orders, and adding polar hydrogens using the Autodock tools(1, 2). Further Structure refinement and optimization were performed with the GROMOS96 implementation by Swiss-PDB Viewer (version19 3.7) to assess the conformational transitions of the FAP enzyme as a target. For ligand preparation, 2D structures of FAPI-MKG and FAPI-46, as the reference ligand, were prepared by ChemSketch software and minimized using the HyperChem8 program through sequential minimization with molecular mechanics (RMSD 0.01 Å and AMBER force field) and semi-empirical methods (PM3). Polar hydrogens and Kollman charges were added using Auto Dock Tools, and the structures were saved in .pdbqt format for docking analysis.

S2.2. Docking Procedure

Molecular docking simulations were conducted using AutoDock Vina, which utilizes a Lamarckian Genetic Algorithm (LGA) for flexible ligand binding (3). A docking grid box with dimensions 126 × 70 × 92 A° was generated, centered at coordinates X=30.668, Y=6.487, Z=15.972 to encompass the active site of FAP. During the docking simulation, the FAP receptor was maintained in a rigid conformation while allowing full torsional flexibility for the ligands. For each ligand, nine docking poses were generated and ranked based on binding energies (kcal/mol). Ligand-receptor interactions, including hydrogen bonding and hydrophobic interactions, were analyzed using AutoDock Tools, UCSF Chimera, and LigPlot $^{++}$. Visualization of hydrophobic contacts and 2D interactions between the ligand and target was conducted using Lig Plot^{++} software (4).

S2.3. Molecular Docking Results

Molecular docking was performed to predict the interactions and binding affinity of ligands of FAPI-MKG and FAPI-46 with the FAP receptor. The crystal structure of FAP was taken from the RCSB Protein Data Bank (PDB ID: 1Z68). Molecular docking simulations were conducted with AutoDock Vina software, and the resulting FAP-ligand complexes were analyzed to determine the optimal binding poses and interaction profiles. The calculated binding energies for the highest-affinity conformations of FAPI-MKG and FAPI-46 with the FAP are summarized in Table S1. Both ligands demonstrated a strong binding affinity to FAP, FAPI-MKG showing a binding energy of -9.8 kcal/ mol and FAPI-46 with a binding energy of -9.7 kcal/ mol. FAP is a 760 amino acid protease consisting of a cytoplasmic N-terminal tail and a β-propeller domain followed by an α/β-hydrolase domain. The extracellular portion, where enzymatic activity occurs, functions as a dimer (5). The catalytic triad, which consists of Ser 624, Asp 702, and His 734, is located at the interface of the β propeller and α/β-hydrolase domains. As shown in Figure S21A, FAPI-MKG demonstrated a strong binding mode with the FAP, mediated by hydrophobic interactions with residues Aap457, Arg402, Tyr458, Phe401, Val356, Pro216, Trp298, Trp295, Trp213, Trp214, Pro157, Trp155, Cys154, Pro107, and Leu105, along with a hydrogen bond formed with His456. These interactions indicate high binding affinity and specificity of FAPI-MKG for the FAP receptor.

S3. Molecular Dynamics Simulation

Molecular dynamics (MD) simulations were carried out using NAMD 3.0, leveraging GPU acceleration to enhance computational performance. The CHARMM36 force field was applied to model interatomic interactions, and trajectory analysis and structural visualization were conducted using Visual Molecular Dynamics (VMD) (6). Following established protocols from prior work (7), the systems underwent a multi-step equilibration process. The receptor was embedded in a TIP3P water box (102 × 115 × 150 Å) and subjected to energy minimization via the steepest descent algorithm. System stability was verified by tracking the root-mean-square deviation (RMSD) and total energy convergence, which plateaued after 50 ns. To enhance statistical reliability, simulations were extended to 200 ns, with three independent replicates performed for each receptor-ligand complex. Data analysis focused on the equilibrated phase, with results averaged across trajectories. For force field consistency, the OPLS-AA parameters were applied alongside the SPC/E water model (8, 9). Ligands were introduced into the pre-equilibrated receptors, and the resulting complexes underwent further relaxation. Temperature regulation (310 K) was maintained using a Langevin thermostat, while pressure was controlled via Langevin piston coupling with a Nosé-Hoover barostat. Long-range electrostatic interactions were computed using the particle-mesh Ewald (PME) method, with a 12 Å cutoff and 1 Å grid spacing (10). Trajectories from the final 100 ns were extracted for quantitative assessment. A custom Tcl script was employed to compute binding free energies and interaction forces. Structural integrity was evaluated through three key metrics: Radius of gyration (RoG) – to assess compactness, RMSD (root-mean-square deviation) – to monitor conformational stability, and RMSF (root-mean-square fluctuation) – to identify flexible regions of the receptor. These analyses provided a detailed perspective on the dynamic behavior of the receptor-ligand systems at equilibrium.

S3.1. Molecular Dynamics Simulation Results

The thermodynamic and structural behavior of ligand-receptor complexes was evaluated through molecular dynamics (MD) simulations. Interaction energies, force distributions, and conformational dynamics were systematically analyzed to compare the binding efficacy of FAPI-46 and FAPI-MKG ligands. Figure S22A shows the non-bonded interaction energies (electrostatic and van der Waals contributions) between receptor residues and ligands, averaged over three independent simulations. Each simulation was repeated three times with different initial conditions, and the values presented are averages from the last 50 ns. As seen in Figure S22A, FAPI-MKG exhibits more favorable (negative) binding energies than FAPI-46, indicating stronger thermodynamic stability. The clear energy minima in FAPI-MKG-bound simulations correspond to key receptor residues, highlighting potential binding hotspots. These findings support the idea that FAPI-MKG forms more stable complexes, likely due to optimized electrostatic complementarity with the receptor. The force distribution map (Figure S22B) highlights key residues involved in attractive (red) and repulsive (blue) interactions with the FAPI-MKG ligand, providing insights into binding specificity and stability. Residues with strong attractive forces (red, -11 to -5 kcal/mol·Å) are crucial for ligand binding. The positive force values (blue, ~2–4 kcal/mol·Å) near ASP731 indicate electrostatic repulsion. Figure S22C tracks the Radius of gyration (RoG) of the receptor alone and when bound to FAPI-MKG or FAPI-46 ligands over a 50 ns simulation. Both ligands induce minor global changes; FAPI-MKG shows a slight expansion (~1 Å) in RoG, but FAPI-46 with a negligible RoG shift (~0.5 Å). The root-mean-square fluctuation (RMSF) (Figure S22D) revealed that FAPI-MKG stabilizes key residues (Ala100,

Ser289, Leu634), reducing their fluctuations and likely enhancing binding affinity. Conversely, residues Gly84 and Ser431 exhibit increased flexibility with FAPI-MKG, potentially reflecting adaptive binding mechanisms.

Table S1: Molecular docking results of the best-docked conformation obtained between the synthesized compound FAPI-MKG, the reference FAPI-46, and FAP as a target using Autodock Vina software.

**Ligands**	**Binding affinity energy(kcal/mol)**
FAPI-MKG	-9.8
FAPI-46	-9.7

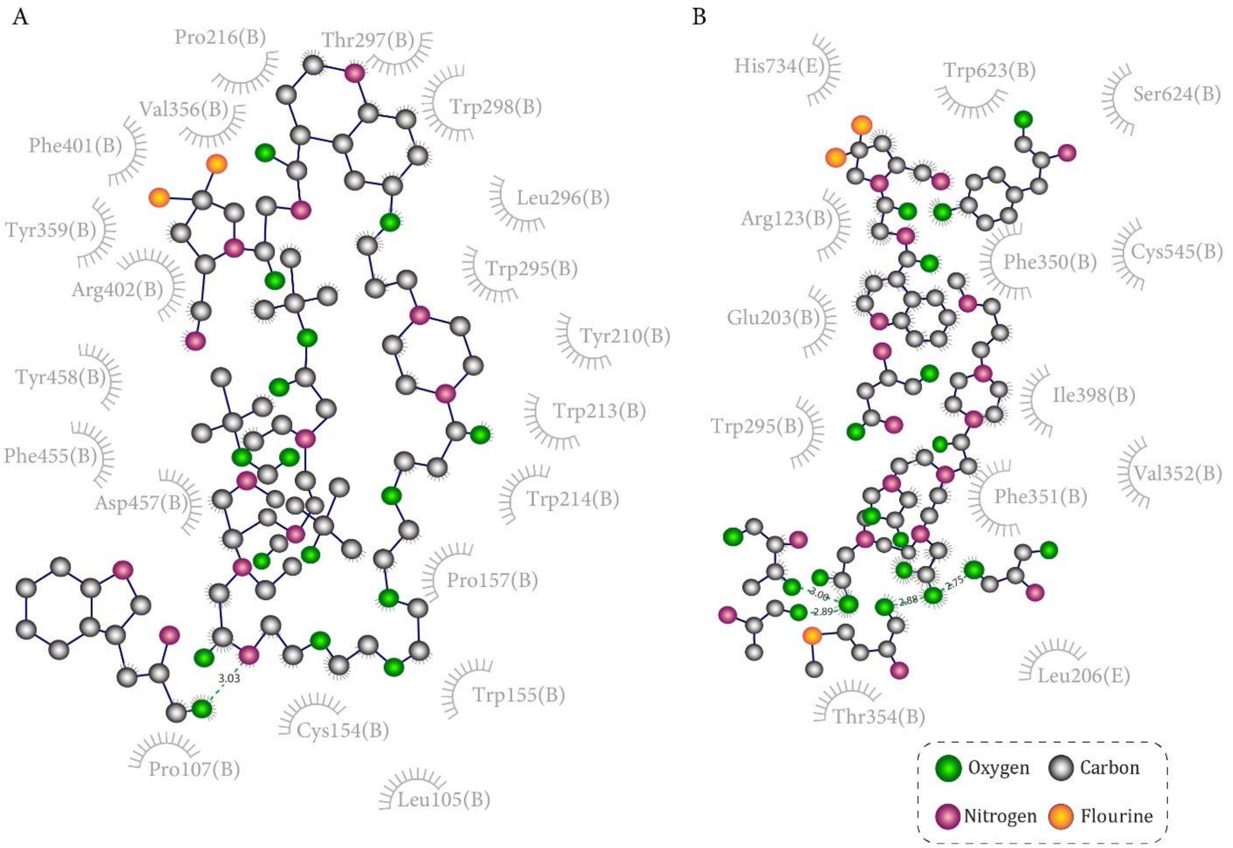

Figure S21. 2D interaction diagrams of (A) FAPI-MKG, and (B) FAPI-46 with FAP receptor (PDB ID: 1Z68)

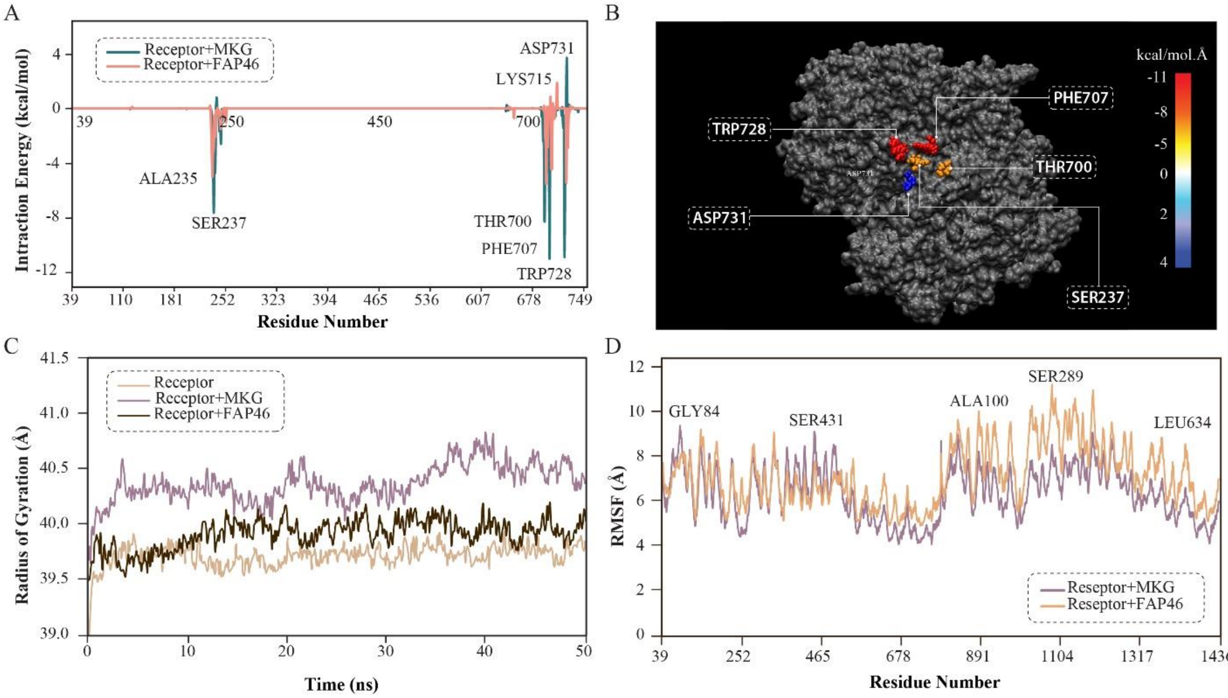

Figure S22. Results of MD simulations, (A) Total interaction energy of receptor-ligand complexes (kcal/mol), averaged across three independent simulations. These values represent non-bonded interactions, including electrostatics, for each receptor residue; (B) Force distribution of FAPI-MKG-ligand on the receptor, averaged over three replicates. Red regions indicate high-force interactions, while gray denotes residues not exposed to the ligand; (C) Radius of gyration (RoG) of receptors with and without ligands, averaged over the final equilibrium frames. Simulations were repeated three times. FAPI-MKG induces an increase in the RoG of approximately 1 Å, suggesting a more open receptor conformation. In contrast, FAPI-46 produces a lower RoG change (~0.5 Å), indicating it has a weaker impact on the receptor's overall structure; (D) Root-mean-square fluctuation (RMSF) analysis of receptor residues in complex with two distinct ligands, FAPI-46 and FAPI-MKG, computed over the final 50 ns from three independent MD trajectories. The plot illustrates residue-specific flexibility, highlighting regions of high conformational variability versus structurally stable elements

The results of molecular docking (Figure S21) and MD simulations (Figure S22) demonstrate that FAPI-MKG exhibits superior binding affinity and specificity for FAP compared to FAPI-46. This enhanced performance is evidenced by more favorable interaction energies, optimized force distributions, and reduced conformational flexibility in critical receptor residues, leading to stronger complex stabilization (Figure S22). The force distribution results (Figure S22B) highlight key residues involved in attractive (red) and repulsive (blue) interactions with the FAPI-MKG ligand, providing insights into binding specificity and stability. Trp728's aromatic side chain probably engages in π-π stacking or hydrophobic interactions with FAPI-MKG, contributing to high binding affinity (11). Tryptophan's rigid structure may also limit ligand mobility, stabilizing the complex. A similar situation applies to Phe707, where hydrophobic and π-stacking interactions dominate. Positioned near FAPI-MKG's hydrophobic core, this residue may help anchor the ligand in the binding pocket. Conversely, polar residues (Ter700 and Ser237) likely form hydrogen bonds with FAPI-MKG, explaining their moderate-force contributions (-5 to 0 kcal/mol·Å). The flexibility of these residues might enable adaptable binding, optimizing ligand orientation. The positive force values (blue, ~2–4 kcal/mol·Å) near Asp731 indicate electrostatic repulsion, likely caused by repelling the negatively charged aspartate side chain with the possible negative charge or dipole of FAPI-MKG. Another possible reason for this behavior could be steric hindrance,

where the bulky carboxylate group of Asp731 may physically block ligand proximity. Radius of Gyration (RoG) analysis helps evaluate both structural compactness and flexibility of the receptor upon ligand binding. A tighter structure (more compactness) may reflect ligand-induced stabilization (reducing entropy and favoring binding) and improved binding pocket integrity (optimizing ligand-receptor contacts). On the other hand, overly rigid conformations might reduce flexibility and hinder functional dynamics (e.g., allosteric signaling). Also, excessive compaction could distort binding sites, weakening interactions. The results of RoG indicate that both ligands induce minor global changes; FAPI-MKG causes a slight expansion (~1 Å) in RoG, implying a more "open" receptor conformation. This structural rearrangement may facilitate stronger binding by exposing additional interaction sites. FAPI-46 shows negligible RoG shifts (~0.5 Å), indicating limited influence on overall receptor topology. The data suggest that FAPI-MKG's superior efficacy arises not only from stronger interactions but also from its ability to modulate receptor flexibility. RMSF, as a residue-specific flexibility test, measures the flexibility of receptor residues during the simulation. Higher RMSF values reflect flexible regions, often associated with binding or conformational change (12). Lower values indicate stable, rigid regions. As Figure S22D shows, FAPI-MKG stabilizes key residues (Ala100, Ser289, Leu634), reducing their fluctuations and likely enhancing the binding affinity. Conversely, Gly84 and Ser431 exhibit increased flexibility with FAPI-MKG, potentially reflecting adaptive binding mechanisms. FAPI-46 confers stability in one receptor chain, while FAPI-MKG dominates in the other, suggesting ligand-dependent modulation of distinct functional regions. These results collectively demonstrate that FAPI-MKG forms more stable and specific complexes with FAP, driven by optimized electrostatic interactions and reduced conformational flexibility at critical binding residues.

References

1. Emamgholipour Z, Dabirian S, Peytam F, Moghadam ES, Firoozpour L, Safavi M, et al. Synthesis, biological evaluation, and in silico study of novel coumarin-quinazoline analogs as potential Anti-Angiogenesis agents. Results in Chemistry. 2024;11:101819.
2. Hassanzadeh L, Erfani M, Jokar S, Shariatpanahi M. Design of a New 99mTc-radiolabeled Cyclo-peptide as Promising Molecular Imaging Agent of CXCR4 Receptor: Molecular Docking, Synthesis, Radiolabeling, and Biological Evaluation. Current radiopharmaceuticals. 2024;17(1):77-90.
3. Behbahani BA, Noshad M, Falah F, Zargari F, Nikfarjam Z, Vasiee A. Synergistic activity of Satureja intermedia and Ducrosia anethifolia essential oils and their interaction against foodborne pathogens: A multi-ligand molecular docking simulation. LWT. 2024;205:116487.
4. Jokar S, Erfani M, Bavi O, Khazaei S, Sharifzadeh M, Hajiramezanali M, et al. Design of peptide-based inhibitor agent against amyloid-β aggregation: Molecular docking, synthesis and in vitro evaluation. Bioorganic Chemistry. 2020;102:104050.
5. Wolf BB, Quan C, Tran T, Wiesmann C, Sutherlin D. On the edge of validation-cancer protease fibroblast activation protein. Mini reviews in medicinal chemistry. 2008;8(7):719-27.
6. Humphrey W, Dalke A, Schulten K. VMD: visual molecular dynamics. J Mol Graph. 1996;14(1):33-8, 27-8.

7. Dashti MA, Mohammad-Aghaie D, Bavi O. Disrupting SARS-CoV-2: molecular dynamics insights into the role of human β-defensin 2 and α-defensin 5 peptides in membrane structure alteration. Chemical Physics Impact. 2024;9:100727.
8. Bavi N, Cox CD, Nikolaev YA, Martinac B. Molecular insights into the force-from-lipids gating of mechanosensitive channels. Current Opinion in Physiology. 2023;36:100706.
9. Bavi N, Martinac AD, Cortes DM, Bavi O, Ridone P, Nomura T, et al. Structural Dynamics of the MscL C-terminal Domain. Scientific Reports. 2017;7(1):17229.
10. Jamali M, Bavi O. Aquaporin channels in desalination: Mechanical properties and operational load analysis. Desalination. 2025;594:118245.
11. Jokar S, Khazaei S, Gameshgoli XE, Khafaji M, Yarani B, Sharifzadeh M, et al. Amyloid β-targeted inhibitory peptides for Alzheimer's disease: current state and future perspectives. Exon Publications. 2020:51-68.
12. Emamgholipour Z, Dabirian S, Peytam F, Saeedian Moghadam E, Firoozpour L, Safavi M, et al. Synthesis, biological evaluation, and in silico study of novel coumarin-quinazoline analogs as potential Anti-Angiogenesis agents. Results in Chemistry. 2024;11:101819.
